# A Gamified Assessment Tool for Antisocial Personality Traits (Antisocial Personality Traits Evidence-Centered Design Gamified): Randomized Controlled Trial

**DOI:** 10.2196/70453

**Published:** 2025-08-25

**Authors:** Yaobin Tang, Yongze Xu, Qunli Zhou, Ran Bian

**Affiliations:** 1 Faculty of Psychology Beijing Normal University Beijing China; 2 Department of Psychology Faculty of Arts and Sciences Beijing Normal University Zhuhai China; 3 Beijing Key Laboratory of Applied Experimental Psychology, National Demonstration Center for Experimental Psychology Education Faculty of Psychology Beijing Normal University Beijing China; 4 Beijing Zhongce Kaiyuan Talent Assessment Technology Co., Ltd Beijing China

**Keywords:** evidence-centered design, gamified assessment, antisocial personality, randomized controlled trial, psychometric validation

## Abstract

**Background:**

The traditional self-report instruments (eg, scales) used to measure antisocial personality traits are characterized by social desirability bias and fail to capture multidimensional behaviors (eg, manipulation and deception).

**Objective:**

This study aimed to develop and validate an evidence-based design for a gamified assessment tool (Antisocial Personality Traits Evidence-Centered Design Gamified assessment tool; ASP-ECD-G) to measure 7 antisocial personality traits (manipulative, callous, deceptive, hostile, risk taking, impulsive, and irresponsible) as defined in the *Diagnostic and Statistical Manual of Mental Disorders, Fifth Edition* (*DSM-5*).

**Methods:**

This research featured a 3-phase evidence-centered design framework. Ontology development (study 1): semistructured interviews were conducted with 9 workplace professionals to translate the *DSM-5* criteria into 24 observable workplace behaviors, which were integrated into a text-based game featuring 10 subscenarios, 34 interactive questions, and logic rooted in logical jumps to simulate real-world decision-making. Model construction (study 2): 6 machine learning models were trained by reference to a set of Personality Inventory for DSM-5 Short Form scores (n=286). The gated recurrent unit model, which uses 1-hot encoding to address nominal response data, was evaluated in terms of the root mean square error (RMSE), mean absolute error, criterion correlation (*r*), and test-retest reliability. Retest reliability was assessed using intraclass correlation coefficients based on 10 participants (1-month interval). Empirical validation (study 3): a 2×2 mixed design (n=148) was used to compare the gamified assessment with questionnaires under conditions involving incentives (ie, situations in which “rational results” led to increased payments).

**Results:**

For model performance, the gated recurrent unit outperformed the alternatives, as indicated by the highest criterion correlation (r=0.850) and the lowest test RMSE (0.273); in particular, it excelled in moderate score ranges (1.5-3, RMSE≤0.377) and in resisting extreme value distortions (3.5-4, RMSE 0.854). Retest reliability was moderate to strong (intraclass correlation coefficients=0.776, *P*=.02). For validation findings, the gamified assessment was associated with higher levels of immersion (mean 7.628 vs 7.216; F147=14.259, *P*<.001) and interest (mean 7.095 vs 6.155; F147=47.940, *P*<.001), although it also elicited stronger negative emotions (mean 4.365 vs 2.473; F147=151.109, *P*<.001). Incentives reduced questionnaire scores (incentivized: 2.066 vs control: 2.201; F1=5.740, *P*=.02) but had no effect on gamified scores (*P*=.71), confirming resistance to manipulation.

**Conclusions:**

By integrating evidence-centered design with gamified workplace simulations, ASP-ECD-G can provide more objective and ecologically valid measurements of antisocial personality traits, thereby supporting both research and organizational practice.

**Trial Registration:**

Open Science Framework (OSF) Registries tvg6x; https://osf.io/tvg6x

## Introduction

### Overview

Antisocial personality (ASP) traits, which are characterized by manipulativeness, callousness, and deceitfulness, entail significant threats to organizational trust, team dynamics, and ethical decision-making [1]. Individuals who exhibit these traits often exploit others for their personal gain, disregard their team responsibilities, and engage in risky behaviors that undermine the long-term organizational health [2,3]. Traditional assessment tools, such as the Psychopathy Checklist–Revised and the Personality Inventory for DSM-5–Short Form (PID-5-SF), rely heavily on self-reports or clinical interviews, which are susceptible to social desirability bias—especially in nonclinical settings, in which individuals may consciously or unconsciously underreport problematic behaviors [4,5]. For example, self-ratings on the PID-5-SF often fail to capture situational impulsivity or deceitfulness, as they lack the ecological validity of real-time behavioral data in high-stakes scenarios [6].

Even structured tools such as situational judgment tests (SJTs), which simulate workplace dilemmas, struggle to address the multidimensional nature of the antisocial traits defined by the *Diagnostic and Statistical Manual of Mental Disorders, Fifth Edition* (*DSM-5*). These tests typically assume item independence and 1-dimensionality, thereby ignoring the interactive effects among different traits (eg, the possibility of manipulativeness and risk taking co-occurring in decision-making) [7]. Specifically, these methods often lack operability and interest in the context of practical application, thus failing to engage participants effectively [1,3,8,9]; accordingly, they are susceptible to subjective influences and the manipulation of results [8,10,11].

### Background

Gamified assessments represent a promising alternative in this context because they embed trait-relevant dilemmas in immersive contexts, in which participants’ choices reflect genuine behavioral tendencies rather than reflective self-evaluations [12]. However, existing gamified tools have not yet been aligned with clinical diagnostic criteria, thus giving rise to a situation of fragmented validation and limited utility in organizational settings that require rigorous psychometric evidence [13-15]. We address this gap by using evidence-centered design (ECD), a systematic methodology that translates theoretical constructs into observable behavioral evidence through a process involving 3 iterative stages: capability modeling, evidence modeling, and task design [16]. In the *capability modeling* stage, we defined target traits (eg, manipulativeness or deceitfulness) based on DSM-5 diagnostic criteria, thus ensuring alignment with clinical and organizational contexts. In the *evidence modeling* stage, we identified behavioral indicators by using empirical methods (eg, expert interviews) to bridge abstract traits with observable decisions in simulated scenarios. In the *task design* stage, we constructed gamified tasks that elicit the target behaviors, particularly using dynamic logic (eg, question‒jump paths) to create immersive, consequence-laden environments in which genuine responses are more likely to emerge.

Although ECD offers significant potential in this context, its application in the field of dark personality traits has not yet been explored in full. This point is especially evident in the context of efforts to operationalize multidimensional constructs such as antisocial behavior.

The ECD framework is a systematic method that can be used to develop assessment tools to construct evidence arguments in game-based assessments beginning in the early stages of assessment by clarifying the evidence needed, and to guide the design and development of the assessment tool accordingly [17]. The ECD assessment has been described as a reasoning process that involves drawing inferences regarding participants’ real-world knowledge and abilities from the limited evidence provided in the test environment. The core of ECD lies in the process of constructing a clear design framework that includes a capability model, an evidence model, and a task model [18]; thus, ensuring that the process of developing the assessment tool remains centered on explicit assessment objectives and evidence requirements [17].

The capability model describes and defines the personality traits to be measured, whereas the evidence model translates the capability model into observable behaviors and performances, including details on how participants’ behaviors in given task contexts reflect the traits included in the capability model [19]. On the basis of the capability model and the evidence model, rules or models are established with the goal of constructing quantitative relationships between the models, which can range from simple scoring rules to complex logic trees or data-driven mathematical models, including machine learning models [20]. The task model describes the specific tasks that participants must complete as part of the assessment tool. These tasks should effectively elicit the target behaviors on the part of participants, thus providing useful evidence.

The ECD approach ensures the systematic and scientific development of assessment tools. This approach can enhance the reliability and validity of assessment tools and reduce assessment errors by establishing a clear design framework and defining evidence needs, as noted in Mislevy et al [17]. Moreover, the ECD approach can respond flexibly to different assessment needs and application scenarios, thus rendering the resulting tools more diverse and broadly applicable. For example, in high-stakes recruitment and selection processes, ECD can facilitate the design of fairer and more effective assessment tools [7].

Our gamified assessment framework, which is anchored in the self-determination theory [21] and flow theory [22], functions based on 3 mutually reinforcing mechanisms: narrative immersion, responsive feedback, and dynamic flow induction. The narrative mechanism involves embedding questions in workplace scenarios (eg, “audit visits” or “human resources [HR] dismissal”) with the aim of allowing participants to adopt a virtual role that is separate from their real-world identity, thus fulfilling the need for autonomy posited by the self-determination theory and reducing individuals’ self-awareness of being assessed, thus mitigating the tendency toward socially desirable responses [23]. The feedback mechanism provides competence-relevant feedback via immediate consequences (eg, dismissal notices in response to dishonest decisions), thus prompting participants to internalize the goal of “surviving” the scenario and to align their responses with in-game logic rather than external judgment; thus, mitigating acquiescence bias [24]. The immersive flow mechanism induces cognitive flow via dynamic path selection (eg, branching storylines based on previous choices), in which context the high cognitive load that results from navigating these paths depletes the mental resources available for response manipulation and effectively “crowds out” deliberate distortion [22].

### Objectives

This study aims to fill this gap by developing and validating the Antisocial Personality Traits Evidence-Centered Design Gamified assessment tool (ASP-ECD-G), which integrates the *DSM-5* criteria with workplace scenarios with the aim of measuring 7 core traits based on behavioral data.

This research was conducted in 3 phases: study 1 involved constructing the assessment ontology based on semistructured interviews; study 2 focused on the development of machine learning models aimed at mapping behavioral data to trait scores; and study 3 entailed validating the tool’s resistance to manipulation and user experience based on a 2×2 mixed experimental design.

## Methods

### Overview

As part of this research, 3 studies were designed to develop the ASP-ECD-G: study 1 involved developing an assessment ontology for ASP as well as constructing the capability model, evidence model, and task model within the framework of ECD; study 2 involved constructing an assessment model that linked the response task model with the capability model based on study 1; and study 3 validated the assessment characteristics of the ASP-ECD-G through a 2×2 mixed experimental design.

### Development of the Assessment Construct

#### Determining the Capability Model

This study used the alternative model for diagnosing ASP disorder from the *DSM-5* [25] as the capability model and integrated it with antisocial behaviors in organizational settings, thereby identifying the 7 behavioral characteristics of ASP provided in Textbox 1.

**Seven behavioral characteristics of antisocial personality.**

1. Manipulativeness: frequent use of charm, glibness, or flattery to influence or control others for personal gain.
2. Callousness: a lack of empathy, which often involves disregarding others’ feelings or problems. When the individual causes harm to others, they express no guilt or remorse and may engage in aggressive and abusive behaviors.
3. Deceitfulness: frequent engagement in fraud against others, misrepresentations of oneself, and embellishments or fabrication of information when it pertains to personal interests.
4. Hostility: persistent and frequent anger, feelings of anger in response to minor slights and insults, and retaliation with harsh, sarcastic, or vengeful behaviors.
5. Risk taking: engagement in potentially dangerous activities without fully considering the consequences, thereby often neglecting personal deficiencies and denying the reality of risks.
6. Impulsivity: rapid responses to immediate stimuli without planning or considering the consequences, and a feeling of difficulty in making and following plans.
7. Irresponsibility: a tendency to shirk one’s duties, commitments, or agreements and to opt out of responsibilities when personal interests are at risk.

#### Constructing the Evidence Model

This study involved semistructured interviews with 9 professionals who had >3 years of work experience, including 1 senior manager from the retail industry; 2 midlevel managers from the telecommunications and smart hardware industries; and 6 frontline employees who were recruited from diverse sectors, such as smart hardware, traditional media, health care, finance, and business consulting. All the interviews were conducted on the web via a third-party platform, and each session lasted 30 to 60 minutes. The interview outline was developed based on the definitions of the 7 behavioral characteristics included in the capability model; its core content is presented in Table 1. Each interview started with warm-up questions with the goals of establishing a trusting relationship, easing tension, and gradually facilitating in-depth discussions while simultaneously collecting relevant background information regarding the participants. The study used the situation, task, action, and result principle to formulate probing questions for each behavioral event, thereby ensuring the completeness and authenticity of the scenario events discussed during the interviews. The interviews concluded when all the questions had been answered, and the interviewees were asked whether they had anything else to add regarding the questions and answers to confirm that no further information was needed.

The interview contents were transcribed from audio to text form. Through manual screening, the interviews were categorized and organized based on the 7 behavioral characteristics outlined in the interview outline. Workplace behaviors often overlap, thereby reflecting multiple characteristics. This is the result of the complex nature of work environments and decision-making processes. Different perspectives and interpretations can lead to varied understandings of individual behavior. For example, a leader berating an employee for the employee’s failure to complete a task because of illness might be viewed as hostile from the leader’s perspective but callous from the employee’s perspective. Accordingly, the study focused on specific scenarios, and 34 scenarios that effectively reflect ASP traits were derived from the 9 interviews. These scenarios are detailed in Multimedia Appendix 1. Specific behavioral characteristics were then extracted from each scenario based on the core content of the question design highlighted in Table 1, in which context, each scenario could include 1 to 7 ASP traits.

**Figure 1:**
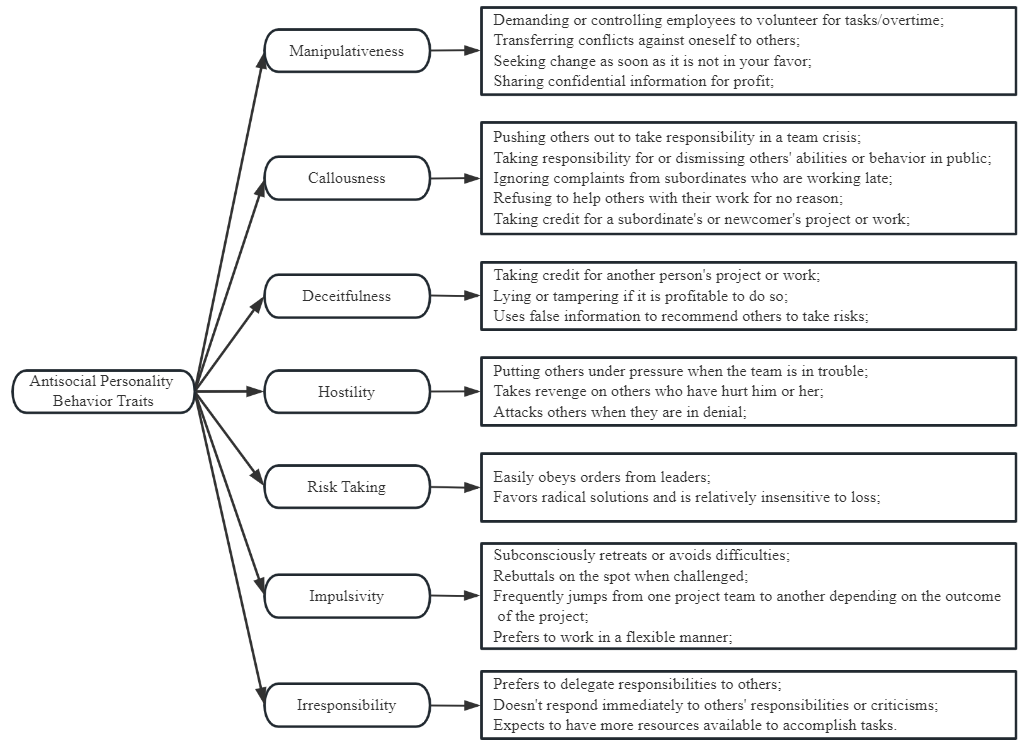
Capability model and evidence model for antisocial personality assessment.

After the 34 scenarios were organized and duplicates removed, 15 unique scenarios remained. The behaviors associated with each role in these scenarios were then extracted and matched with the 7 ASP traits, thus facilitating the identification of 24 typical workplace behaviors. The evidence model was then constructed, as illustrated in Figure 1.

**Table 1:** Semistructured interview outline used to define antisocial personality behaviors according to the DSM-5a with workplace professionals (n=9).

Behavioral characteristics	Core content of the question design
Warm-up question	“Briefly introduce your career experiences in chronological order”
Manipulativeness	Not following company rules, regulations, or laws
Deceitfulness	Intentionally hiding a great deal of information; delivering or reporting false information
Impulsivity	Making workplace decisions driven by emotions or motives without fully considering relevant information
Hostility	Expressing strong hostility, anger, or dissatisfaction, and proactively attacking others
Risk taking	Demonstrating indifference to workplace safety and regulations, including taking actions that pose potential risks to both individuals and teams
Irresponsibility	Displaying dereliction of duty or irresponsibility, leading to an inability to complete one’s tasks on time
Callousness	Showing a lack of remorse for wrongdoings and an unwillingness to admit one’s mistakes and improve
Probing questions	Probing and supplementing relevant information based on the STAR^b^ principle

^a^DSM-5: Diagnostic and Statistical Manual of Mental Disorders, Fifth Edition.

^b^STAR: situation, task, action, and result.

#### Constructing Assessment Tasks

We operationalized these behavioral characteristics in a game setting by incorporating 34 representative workplace behaviors into an interactive game consisting of 10 detailed subscenarios. We assigned each virtual character a basic name, departmental affiliation, and personal background information to enhance the realism of the game. In addition, we carefully designed transitions between scenes to ensure that the narrative flow was coherent. The relationships between each game scenario and the corresponding behavioral characteristics are presented in Table 2.

**Figure 2:**
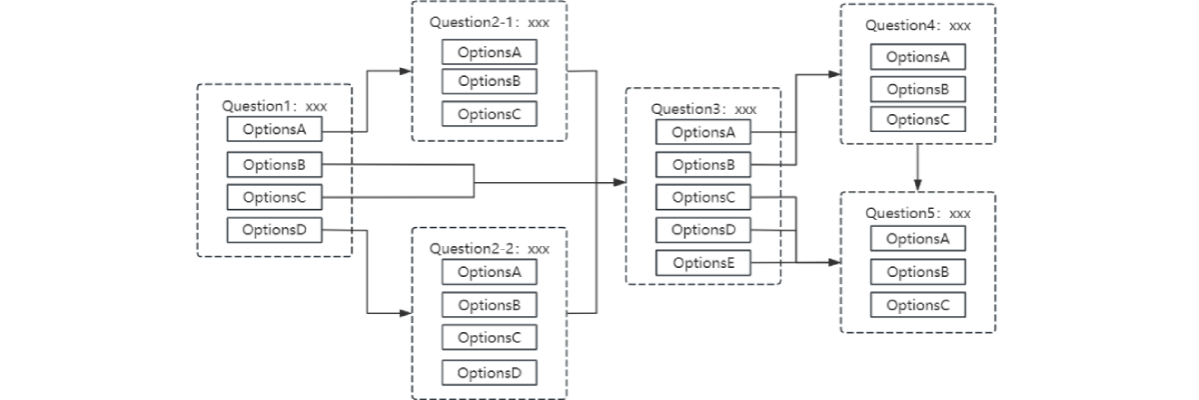
Logical roadmap for the gamified assessment tool.

Three psychology experts with more than 3 years of workplace experience reviewed and revised the questions, options, and jump relationships based on the capability and evidence models until they reached a consensus. The finalized game consisted of 34 questions with 115 options (2-5 options per question), including 13 questions featuring logical jump relationships across 6 scenarios. This jump logic took 2 forms: question jump (eg, a situation in which choosing Q1-A jumps to Q3, whereas other options proceed to Q2) and progressive (eg, a situation in which Q1/Q2-D jumps directly to Q4). The logical design is illustrated in Figure 2. Examples of the game’s item presentation are provided in Multimedia Appendix 2.

**Table 2:** Game scenarios and corresponding antisocial traits in the ASP-ECD-G^a^ design (10 game scenarios and 34 workplace behavior characteristics).

Game scenario	Workplace behavior characteristics	Antisocial personality traits
Project and proposal decision	Preference for radical proposals and relative insensitivity to losses	Risk taking
Proposal reporting	Taking credit for others’ proposals or achievements Seizing subordinates’ or newcomers’ proposals or achievements	Callousness and deceitfulness
Project initiation meeting	Retaliation against others who have harmed the focal individual Proactive attacks when criticized Immediate rebuttal when questioned Publicly criticizing or denying others’ abilities or actions Shifting blame for personal conflicts to others	Hostility, impulsivity, and manipulativeness
Helping others	Unreasonably refusing to help others at work	Callousness
Delivering results	Demanding or requiring employees to take on tasks or overwork Shifting blame for personal conflicts to others Ignoring complaints regarding overtime from subordinates Failing to respond to criticism during critical periods	Manipulativeness, callousness, and irresponsibility
Altering parameters	Readily obeying orders from superiors Lying or altering information for personal gain	Risk taking and deceitfulness
Emergency situations	Frequently switching project groups or teams based on outcomes A preference for flexible and changeable work Pushing others to take responsibility during team crises Demanding or requiring employees to take on tasks or overwork Allowing others to bear pressure in situations featuring team difficulties Expecting more resources to complete tasks Instinctive retreat, or avoidance, in response to difficulties	Impulsivity, callousness, manipulativeness, and irresponsibility
Information leaks	Sharing confidential information for personal gain Lying or altering information for personal gain Recommending that others should take risks based on false information	Manipulativeness and deceitfulness
Audit visits	Retaliation against others who have harmed the focal individual Sharing confidential information for personal gain Shifting blame for personal conflicts to others Pushing others to take responsibility during team crises Lying, or altering information, for personal gain Preference for radical proposals and relative insensitivity to losses Expecting more resources to complete tasks	Hostility, manipulativeness, callousness, deceitfulness, impulsivity, and irresponsibility

^a^ASP-ECD-G: Antisocial Personality Traits Evidence-Centered Design Gamified assessment tool.

### Assessment Model Construction

#### Dependent Variable Acquisition

During the process of model training for the ASP-ECD-G, we used participants’ scores on ASP items drawn from the simplified Chinese version of the DSM-5 Personality Inventory (PID-5-SF) [5] as labels for the training set (hereafter referred to as ASP scores). The PID-5-SF has exhibited good reliability and validity in previous research, as indicated by a Cronbach α coefficient of 0.916. The specific items are detailed in Multimedia Appendix 3.

#### Independent Variable Encoding Methods

We were inspired by traditional SJTs to assign scores to each option, although doing so exclusively based on fixed rules was impractical. The scoring rules were defined without consideration of the influences of prior questions, thus allowing them to serve as a comparative scheme to contrast this method with the 1-hot encoding approach in computer science.

Expert scoring involves assigning values to each option based on the number of ASP traits that it reflects (eg, 5 points for 1 option that reflects 5 out of 7 traits). Three experts assisted in the scoring process, and only the consensus scores obtained after multiple discussions were used. Because of varying question completion rates, the input of each participant was reshaped into a 34×2 matrix, in which context the figure of 34 represented the total number of questions, and the figure of 2 indicated question completion status and scores, for example, (0,0) for unanswered questions and (1,2) for a score of 2. The final training dataset consisted of a 286×34×2 matrix based on data obtained from 286 participants.

In this study, categorized option data that lacked ordinal information were processed via the 1-hot encoding approach. Specifically, each question was encoded as a 5-dimensional vector: for questions that featured fewer than 5 options (eg, Q1 featured only 4 options), zeros were added to the fifth dimension; when option A was selected, the first dimension was set to 1, and the remaining dimensions were set to 0; for unanswered questions, a 5D vector containing all zeros was generated. Ultimately, the training dataset is represented in the form of a 286×34×5 3D matrix.

#### Selection of Research Models

In our exploration of how game behaviors influence ASP traits, the linear regression (LR) model provides a preliminary framework to analyze the linear relationship between participants’ behavioral data and their levels of antisocial traits. However, deeper insights into decision-making require more complex machine learning methods.

The random forest (RF) model, which involves constructing multiple decision trees and aggregating predictions, captures nonlinear data relationships more accurately but may not fully reveal the interactions among different options. Artificial neural networks (ANNs), which are capable of simulating brain processing, excel at extracting nonlinear patterns when each game option is treated as an independent variable featuring interactive effects.

Because of the sequential and skip-based nature of the questions considered in this context, participant choices form time-series data, thus rendering recurrent neural networks (RNNs) suitable for capturing dynamic changes. However, standard RNNs face gradient issues involving long sequences. The gated recurrent unit (GRU) and long short-term memory (LSTM) models address this issue via gating mechanisms: GRUs simplify the structure by merging hidden states with gates, whereas LSTM models use 3 gates to manage information flow.

Previous research has relied on statistical scoring rules, but the question structure of the ASP-ECD-G has made it challenging for experts to assign consistent scores to identical options or scenario paths. The complexity of behavioral data renders expert scoring impractical. Thus, this study prioritized statistical and machine learning models (ie, LR, RF, ANN, RNN, GRU, and LSTM) for the analysis of gamified data, thus establishing a balance between computational efficiency and interpretability.

#### Model Training Process

Model training was performed with the assistance of the Adam optimization algorithm and the dropout technique, with the aim of optimizing performance by adjusting the batch size, number of epochs, and fine-tuning model complexity. Standard hyperparameters were established for different models: in the RF approach, n_estimators and max_depth were tuned to balance performance and prevent overfitting; in ANN, the number of layers or neurons and activation function were optimized with the goal of improving learning; and in RNN, GRU, and LSTM, hidden size, the number of units, and dropout were used to affect learning or memory and generalization, and the learning rate was carefully chosen for the optimizer. Details regarding the parameters are presented in Table 3.

**Table 3:** Model hyperparameter tuning results.

Model type	Hyperparameters	
	Raw score assignment	1-hot encoding of raw scores
LR^a^ model	None	None
RF^b^ model	n_estimatorsc=32 random_state=2024 max_depthd=4 min_samples_splite=5 min_samples_leaff=1 max_featuresg=“log2”	n_estimators=5 random_state=2024 max_depth=6 min_samples_split=2 min_samples_leaf=1 max_features=“log2”
ANN^h^ model	Layers: 2 Neurones: 32 Activation: “relui” Dropout: 0.5 Optimizer: Adam (lrj=0.001) Loss: “msek” Epochs: 50 Batch size: 10	Layers: 2 Neurones: 16 Activation: “relu” Dropout: 0.5 Optimizer: Adam (lr=0.001) Loss: “mse” Epochs: 100 Batch size: 10
RNN^l^ model	Layers: 1 (RNN, 32 units) Activation: “relu” Dropout: 0.4 Optimizer: Adam Loss: “mse” Epochs: 100 Batch size: 10	Layers: 1 (RNN, 32 units) Activation: “relu” Dropout: 0.2 Optimizer: Adam Loss: “mse” Epochs: 100 Batch size: 50
GRU^m^ model	Layers: 1 (GRU, 64 units) Activation: “relu” Optimizer: Adam (lrn=0.01) Loss: “mse” Epochs: 100 Batch size: 50	Layers: 1 (GRU, 32 units) Activation: “relu” Optimizer: Adam (lr=0.001) Loss: “mse” Epochs: 200 Batch size: 10
LSTM^o^ model	Layers: 1 (LSTM, 16 units) Activation: “relu” Optimizer: Adam Loss: “mse” Epochs: 200 Batch size: 50	Layers: 1 (LSTM, 64 units) Activation: “relu” Optimizer: Adam Loss: “mse” Epochs: 200 Batch size: 10

^a^LR: linear regression.

^b^RF: random forest.

^c^n_estimators: number of estimators.

^d^random_state: random state.

^e^max_depth: maximum depth.

^f^min_samples_split: minimum samples split.

^g^min_samples_leaf: minimum samples per leaf.

^h^max_features: maximum features.

^i^ANN: artificial neural network.

^j^relu: rectified linear unit.

^k^lr: learning rate.

^l^mse: mean squared error.

^m^RNN: recurrent neural network.

^n^GRU: gated recurrent unit.

^o^LSTM: long short-term memory.

#### Model Evaluation Metrics

The predictive performance of the ASP-ECD-G model was evaluated by reference to common metrics (ie, root mean square error [RMSE], mean absolute error [MAE], and criterion correlation, r) with the goal of assessing its accuracy and correlation with reference results. Lower RMSE or MAE values indicate higher levels of accuracy, whereas an *r* value close to 1 signifies a strong positive correlation, thus validating the predictive ability of the model.

As part of this study, 10 participants who met the same criteria as those used in study 2 were recruited to assess the test-retest reliability of the ASP-ECD-G tool. The gamified assessment was administered to these participants twice, with a 1-month interval between the 2 administration periods. The sample size of 10 is in line with relevant guidelines for reliability testing in small-scale validation studies, which focus on within-individual consistency over time. The test-retest design focused on evaluating the stability of behavioral responses to identical scenarios. In both administrations, all 34 interactive questions and logical jump paths were retained.

#### Participants

As part of this study, the Credamo platform (Beijing Yishu Mofa Technology Co, Ltd) was used to digitalize the gamified assessment questionnaire. The questionnaire included demographic variables (ie, gender, age, highest level of education, marital status, employment status, and position), as well as the gamified assessment tool and the ASP questionnaire.

The sampling criteria used for this study were as follows: (1) participants aged ≥18 years; (2) participants who had at least 1 job or internship experience; and (3) nonpsychology majors.

Participants who did not meet these criteria were excluded via the platform’s custom filtering mechanism. The questionnaire was distributed primarily via online social networks such as WeChat groups and Moments. A total of 291 eligible questionnaires were ultimately collected for this study. After further filtering based on lie detection items, 286 valid questionnaires were obtained.

The descriptive statistics of the sample referenced in study 2 are presented in Table 4. Regarding gender distribution, this study included 166 (58%) male participants and 120 (41.9%) female participants out of the 286 participants. With regard to age distribution, the study included 59.4% (170/286) of the participants aged between 23 and 28 years, followed by 26.6% (76/286) aged between 19 and 22 years. For participants’ levels of education, the sample predominantly included individuals with undergraduate degrees (234/286, 81.8%), followed by those with master’s degrees (36/286, 12.6%). Regarding marital status, 68.9% (197/286) of the participants were single. With respect to employment status, 62.9% (180/286) of the participants were employed at the time of this study, and 24.1% (69/286) were serving as interns. For participants’ positions, 62.6% (179/286) of the participants were junior employees, followed by 21% (60/286) who occupied junior management positions.

**Table 4:** Demographic characteristics of the study 2 participants (n=286).

Variable and category			Sample, n (%)
**Sex**			
	Male	166 (58)	
	Female	120 (42)	
	Intersex	0 (0)	
**Age (y)**			
	19-22	76 (26.6)	
	23-28	170 (59.4)	
	29-35	36 (12.6)	
	36-45	4 (1.4)	
**Highest level of education**			
	High school or vocational school	2 (0.7)	
	Associate degree	13 (4.5)	
	Bachelor’s degree	234 (81.8)	
	Master’s degree	36 (12.6)	
	Doctorate	1 (0.3)	
**Marital status**			
	Single	197 (68.9)	
	Engaged	11 (3.8)	
	Married, no children	39 (13.6)	
	Married, with children	30 (10.5)	
	In a relationship	9 (3.1)	
**Employment status**			
	Employed	180 (62.9)	
	Interning	69 (24.1)	
	Formerly employed	13 (4.5)	
	Never employed	21 (7.3)	
	Other	3 (1)	
**Position**			
	Junior employee	179 (62.6)	
	Junior management	60 (21)	
	Midlevel management	30 (10.5)	
	Senior management	12 (4.2)	
Other			5 (1.7)
Total			286 (100)

### Analysis of Assessment Properties

#### Study Design

We built on study 2 by incorporating items used to measure pleasure, interest, positive emotions, negative emotions, and immersive experience into the gamified assessment tasks and the ASP questionnaire. These items are scored on a scale from 1 to 9, in which context higher scores indicate stronger experiences. The specific items are detailed in Multimedia Appendix 3.

We used a 2×2 mixed experimental design, in which the assessment format (ie, gamified assessment vs questionnaire assessment) was used as the within-subject variable, and participant incentive (ie, with vs without) was used as the between-subject variable. This study aimed to investigate the impacts of participant incentives on individual performance across different assessment formats via gamified motivational mechanisms.

After the participants completed the questionnaire, they were asked the following question: “If you were to participate in a company’s new employee psychological test, which method would you prefer?” This question aimed to gauge their future willingness to use gamified assessment versus traditional questionnaire assessment.

Participant incentives were introduced as an external motivator to stimulate achievement motivation, in line with previous research that has linked ASP traits to instrumental rationality, that is, the prioritization of strategic self-interest over social norms [26,27]. Individuals who exhibit high levels of manipulativeness or deceitfulness often engage in utility-maximizing behaviors, thereby justifying their self-serving actions as rational responses to their perceived circumstances [26]. Individuals who exhibit stronger tendencies toward an ASP rationalize their unethical behavior as a necessary form of self-preservation, reframing it as a logical choice rather than an antisocial tendency [27].

Rather than directly incentivizing “antisocial tendencies,” which carry a strong social stigma, this study framed the incentive based on “rationality,” a positively valenced construct that aligns with participants’ self-perceived strategic competence. While external incentives can enhance individuals’ task focus in challenging situations, they may also lead to deceptive self-presentation [28]. The design of this study was based on the hypothesis that this framing would enhance social desirability effects in questionnaires, in which context participants could adjust their responses consciously. Moreover, participants’ gamified assessment scores, which were based on the behavioral choices made in the context of immersive scenarios, were expected to remain unaffected.

The experimental condition involved an increase in the participation payment (from 9-15 RMB [US $1.25-$2.1]) in exchange for “rational results,” without defining specific criteria; this approach aimed to mimic the context of real-world strategic self-presentation (eg, job applicants tailoring answers with the goal of appearing competent). Participants in the control group completed the gamified and questionnaire assessments in the usual manner, whereas those in the experimental group were presented with an incentive: “This study hopes that you can be rational. If your results show that you are rational, we will increase your participation payment (from 9 to 15 yuan).”

#### Participants

The participants were selected based on criteria that were consistent with those used in study 2, and the questionnaires were distributed via the Credamo platform. We used GPower (version 3.1) software to calculate the required sample size to ensure sufficient statistical power for the repeated-measures ANOVA conducted as part of this 2×2 mixed experimental design. Regarding the mixed design featuring 2 between-subject and 2 within-subject assessments, we tested the main effects for both factor types. The analysis, which was based on a medium effect size (f=0.25), α=0.05, power=0.8, and a within-subject correlation assumption of 0.5, indicated minimum sample sizes of 98 for between-subject effects, 34 for within-subject effects, and 68 for interaction effects.

We collected 200 questionnaires across 4 groups with the goal of maximizing participation. A total of 148 valid responses remained after a screening process involving lie detection items. Each group (ie, the group with incentives and the group without incentives) included 74 participants.

Descriptive statistics concerning the sample are presented in Table 5. Of the 148 participants, 94 (63.5%) were female and 54 (36.5%) were male. The participants were predominantly aged between 29 and 35 years (54/148, 36.5%), followed by those aged between 23 and 28 years (45/148, 30.4%). Most participants had obtained bachelor’s degrees (98/148, 66.2%), followed by those who had obtained associate degrees (19/148, 12.8%). The majority of participants (95/148, 64.2%) were married with children. Regarding participants’ employment status, of the 148 participants, 143 (96.6%) were employed, whereas 5 (3.4%) were serving as interns. The participants’ job positions spanned from entry level to senior management, although entry-level employees represented the largest group (59/148, 39.9%).

**Table 5:** Demographic characteristics of the participants in study 3 (n=148).

Variable and category			Participants, n (%)
**Sex**			
	Male	54 (36.5)	
	Female	94 (63.5)	
	Intersex	0 (0)	
**Age range (y)**			
	19-22	11 (7.4)	
	23-28	45 (30.4)	
	29-35	54 (36.5)	
	36-45	27 (18.2)	
	46-50	11 (7.4)	
**Education**			
	Junior high school	3 (2)	
	High school or vocational	7 (4.7)	
	Associate degree	19 (12.8)	
	Bachelor’s degree	98 (66.2)	
	Master’s degree	21 (14.2)	
**Marital status**			
	Single	40 (27)	
	Engaged	1 (0.7)	
	Married, no children	9 (6.1)	
	Married with children	95 (64.2)	
	In a relationship	3 (2)	
**Employment**			
	Employed	143 (96.6)	
	Intern	5 (3.4)	
**Position**			
	Entry-level	59 (39.9)	
	Junior management	36 (24.3)	
	Middle management	42 (28.4)	
	Senior management	11 (7.4)	

### Ethical Considerations

This study was approved by the ethics committee of Beijing Normal University (approval BNU202503310097), thus ensuring compliance with ethical guidelines. All the participants provided written informed consent. The informed consent form detailed the purpose of the study, the procedures used in this research, and the participants’ right to withdraw from the study without penalty; furthermore, it noted that the use of data was limited to the purposes of the study and that the data collected as part of this research would be anonymized for analysis. The participants’ privacy was protected via the deidentification of personal data, the secure storage of such data on password-protected computers, and the aggregated reporting of study results. All the participants received a basic participation payment of RMB 9, and those in the experimental group in study 3 received an additional RMB 6 as a reward for “rational outcomes”; these payments were provided via a digital platform and were not linked to trait scores to prevent bias. No identifiable information or images concerning participants are included in the manuscript or the multimedia appendices; thus, no additional consent for personal identification was needed. These measures are consistent with relevant ethical standards for informed consent, confidentiality, and participant well-being as outlined in the institutional and international research guidelines.

## Results

### Overview

**Figure 3:**
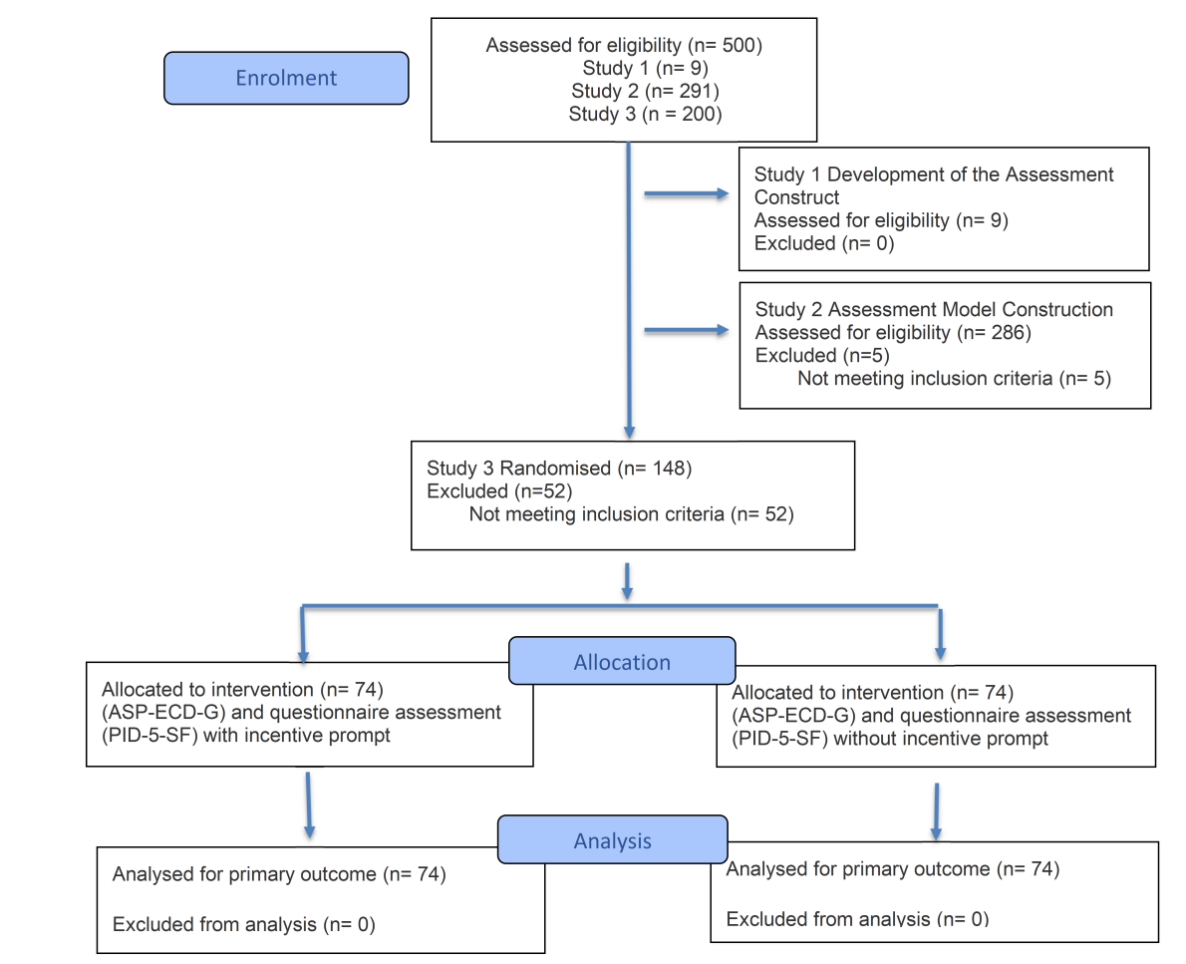
Research design flow diagram of three studies.

Study 1 involved semistructured interviews with 9 professionals (1 senior manager, 2 midlevel managers, and 6 frontline employees) with >3 years of experience; each interview took 30-60 minutes. For study 2, the Credamo platform was used to distribute digital questionnaires (demographics, gamified assessment, and ASP) to participants (aged ≥18 years, with work or internship experience, nonpsychology majors); 291 responses were collected, 286 of which were valid after lie detection. Recruitment for study 3 also used Credamo (with the same criteria as study 2); we collected 200 questionnaires, 148 of which were valid (n=74 per group with or without incentives after lie detection). The design process is shown in Figure 3.

### Development of the Assessment Construct

The ASP-ECD-G is presented as a text-based game provided on a questionnaire platform. The storyline of the ASP-ECD-G simulates a workplace scenario in which players play the role of an employee who has just completed a 1-month probation period and is facing the challenge of securing a permanent position. Players must solve various workplace problems, including conflicts with colleagues, crisis management, ethical dilemmas, and team performance management. Each choice made by the player influences the development of the plot; however, the ultimate outcome involves the player receiving a dismissal notice from human resources.

The design of the ASP-ECD-G incorporates 3 game elements: narrative, immersion, and feedback. The narrative is integrated into the assessment content and is presented in the form of a workplace story in which participants are required to immerse themselves to answer questions. For each question, the game characters provide preset feedback based on the player’s choices, thereby driving the plot forward. Players must select the most suitable options from the choices available within the scenario, and they do not have the ability to save their progress or exit the game at a midway point. Incorrect selections can be rectified by returning to the previous page to reanswer the questions. The presentation of the ASP-ECD-G is illustrated in Multimedia Appendix 4.

The ASP-ECD-G, similar to SJTs, is rooted in real-world scenarios and combines questions with options for assessment. However, in comparison with traditional scales and SJTs, the gamified assessment tool developed as part of this study exhibits 3 distinctive features. First, the scenarios included in the ASP-ECD-G follow a narrative sequence, and the options are characterized by logical transitions, thus violating the independence assumption among the items. Second, because of the logical transitions between questions, different participants may experience varying numbers of scenes during the course of the gamified assessment. Third, each question included in the ASP-ECD-G reflects 1 or more behavioral traits, thereby violating the 1-dimensionality assumption.

Because of these differences in data types, item interdependencies, and dimensional assumptions between the multidimensional nature of the ASP-ECD-G and traditional assessment methods, this study details the findings obtained via multidimensional validation analyses—in which item response theory is used alongside the nominal response model—in Multimedia Appendix 5 for the benefit of readers seeking in-depth insights. This study highlights the fact that these features make the assessment process of the ASP-ECD-G resemble a cohesive story rather than a collection of isolated questions. The ASP-ECD-G aims to replicate real workplace scenarios as closely as possible, and the logical transitions between different options enhance the coherence and immersion of the presented scenario for participants during the assessment process.

### Assessment Model Construction

#### Results of the Expert-Assigned Coding

The expert panel often found it difficult to reach a consensus during the process of scoring game options, thus leading to instability in the scoring results. The task of explaining the sources of differences in scores among participants was challenging, even if scores could be assigned to all paths. The core issue pertained to the task of determining whether different participants who chose the same option in response to the same question should be assigned the same score. Further consideration revealed that treating participants’ responses as a path made it difficult to account for the complexity and extensive information involved in this process. This situation led to instability in the scoring results, as the experts faced similar challenges in the process of scoring both question options and different paths within the same scenario.

After a fixed random seed was determined, the performances of different models on the same dataset were subjected to a comprehensive comparison. The evaluation included the RMSE and MAE for the training and testing sets, as well as the correlation (r) between the predicted and reference results on the testing set; this evaluation aimed to assess the performance of each model.

As indicated in Table 6, none of the models exhibited significant overfitting or underfitting following parameter tuning. Among the testing set results, the GRU model exhibited the best performance, as indicated by RMSE and MAE values of 0.380 and 0.313, respectively, and a correlation of 0.676, thus indicating a high level of consistency between the predicted and observed results.

The study further evaluated the performance of each model across different ASP score ranges in the testing set to assess the stability of the predictions. These results are presented in Table 7.

**Table 6:** Expert-assigned coding model performance for the prediction of antisocial personality scores (n=286; training–test split, 7:3).

Dataset and evaluation metric		LR^a^ model	RF^b^ model	ANN^c^ model	RNN^d^ model	GRU^e^ model	LSTM^f^ model
**Training set**							
	RMSE^g^	0.415	0.432	0.438	0.436	0.363	0.447
	MAE^h^	0.332	0.357	0.343	0.353	0.288	0.356
	r^i^	0.637	0.694	0.601	0.598	0.762	0.559
**T** **esting set**							
	RMSE	0.453	0.418	0.462	0.436	0.380	0.433
	MAE	0.356	0.336	0.362	0.340	0.313	0.344
	r	0.510	0.626	0.529	0.523	0.676	0.567

^a^LR: linear regression.

^b^RF: random forest.

^c^ANN: artificial neural network.

^d^RNN: recurrent neural network.

^e^GRU: gated recurrent unit.

^f^LSTM: long short-term memory.

^g^RMSE: root mean square error.

^h^MAE: mean absolute error.

^i^r represents the correlation between the predicted results and the reference results for the best-performing model.

**Table 7:** RMSE^a^ values of expert-assigned coding models across different ASP^b^ score ranges (test set, N=59)^c^.

ASP score range	Sample size	LR^d^ model	RF^e^ model	ANN^f^ model	RNN^g^ model	GRU^h^ model	LSTM^i^ model
1-1.5	1	0.594	0.757	0.314	0.760	0.483	0.826
1.5-2	13	0.243	0.325	0.297	0.350	0.318	0.252
2-2.5	23	0.360	0.243	0.387	0.278	0.356	0.281
2.5-3	15	0.451	0.402	0.470	0.353	0.377	0.466
3-3.5	6	0.686	0.682	0.478	0.727	0.465	0.627
3.5-4	1	1.537	1.396	1.380	1.518	.854	1.492

^a^RMSE: root mean square error.

^b^ASP: antisocial personality.

^c^No samples featuring ASP scores of ≥4 were included in the testing set.

^d^LR: linear regression.

^e^RF: random forest.

^f^ANN: artificial neural network.

^g^RNN: recurrent neural network.

^h^GRU: gated recurrent unit.

^i^LSTM: long short-term memory.

An analysis of model performance across the range of different ASP scores revealed that the LR model struggled to address complex relationships, as indicated by its high RMSE in mid-to-high ranges (eg, 2.5-3: 0.451; 3-3.5: 0.686), although it exhibited a moderate level of performance in the 1.5 to 2 range (0.243). The RF model excelled in the 1.5 to 2.5 range (RMSE 0.243-0.325) but exhibited instability in cases involving extreme scores (3.5-4: 1.396). The ANN model maintained a low RMSE in lower ranges (1-2.5:≤0.387) but showed increased errors in higher ranges (2.5-3: 0.470; 3-3.5: 0.478). The RNN model exhibited midrange consistency (1.5-3: 0.278-0.353) but was characterized by the highest RMSE in the 3.5 to 4 range (1.518). The GRU model exhibited notably balanced performance: the lowest RMSE was observed in the 1.5 to 2 range (0.318), moderate values were observed in the middle range (2-2.5: 0.356; 2.5-3: 0.377), and relatively controlled errors were observed at the extremes (3.5-4: 0.854). While the LSTM model matched the GRU in the 1.5 to 2 range (0.252 vs 0.318), it exhibited the worst performance in high ranges (3.5-4: 1.492), thus highlighting its inferior handling of sparse data. Given its balanced performance and resilience to small samples, the GRU model was optimal for the prediction of ASP scores.

**Figure 4:**
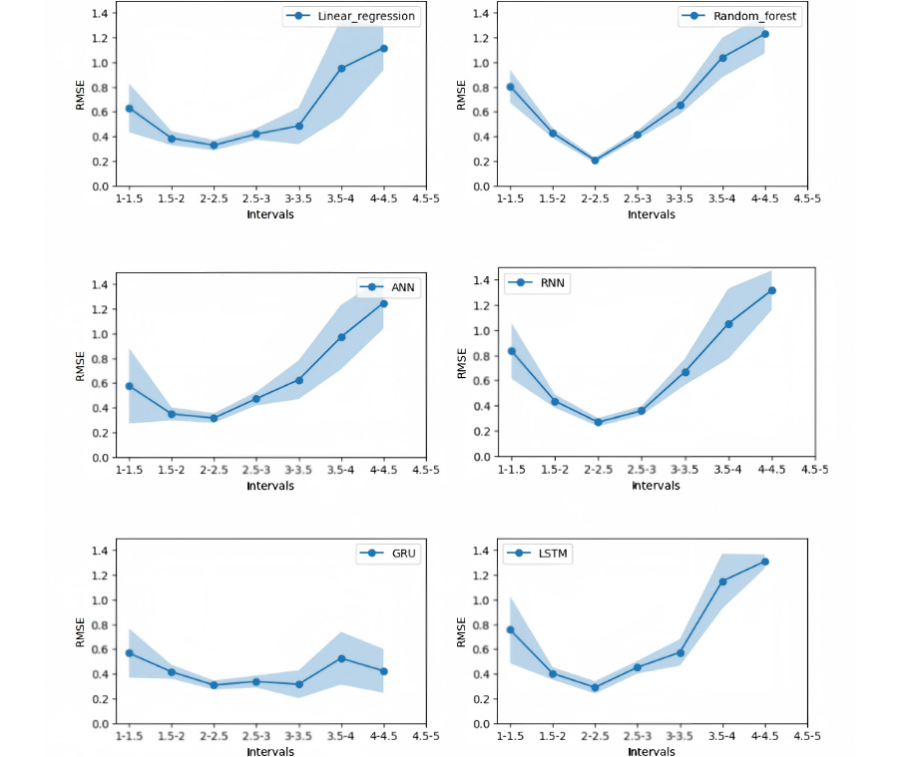
Root mean square error (RMSE) variations across 100 random testing set selections for different models (expert-assigned coding). RMSE means are shown with SD bands. ANN: artificial neural network; GRU: gated recurrent unit; LSTM: long short-term memory; RNN: recurrent neural network.

This study used 100 different random seeds to facilitate the random sampling of the test set with the goal of exploring the robustness of each model in greater depth. In these 100 experiments, changes in the RMSE values were observed across different ASP score ranges. The results are presented in Figure 4. The line shown in this figure indicates the average value across 100 iterations, and the light blue area represents the average plus or minus 1 SD. These points also apply to Tables 4 and 5.

These results indicate that, even after 100 iterations, the GRU model consistently outperformed the other models and exhibited higher levels of stability.

#### Results of 1-Hot Encoding

We used the same fixed random seed to perform a comprehensive comparison of the predictive performance of different models on the same dataset.

As indicated in Table 8, no significant overfitting or underfitting was observed after the parameters were tuned for all the models. We further evaluated the performance stability of each model across different ASP score ranges in the test set, as detailed in Table 9.

**Table 8:** Model performance for antisocial personality score prediction using 1-hot encoding (n=286, 34×5–dimensional encoding).

Dataset and metric			LR^a^ model		RF^b^ model		ANN^c^ model		RNN^d^ model		GRU^e^ model		LSTM^f^ model
**Training set**													
	RMSE^g^	0.336		0.384		0.358		0.387		0.303		0.359	
	MAE^h^	0.269		0.301		0.285		0.299		0.230		0.270	
	r^i^	0.782		0.744		0.770		0.700		0.789		0.748	
**Test set**													
	RMSE	0.638		0.401		0.406		0.420		0.322		0.328	
	MAE	0.426		0.317		0.323		0.322		0.251		0.233	
	r	0.492		0.619		0.677		0.581		0.808		0.760	

^a^LR: linear regression.

^b^RF: random forest.

^c^ANN: artificial neural network.

^d^RNN: recurrent neural network.

^e^GRU: gated recurrent unit.

^f^LSTM: long short-term memory.

^g^RMSE: root mean square error.

^h^MAE: mean absolute error.

^i^r represents the correlation between the predicted results and the reference results for the best model.

**Table 9:** RMSE^a^ values of 1-hot encoding models across different ASP^b^ score ranges (test set, N=59, 5-dimensional vectorization)^c^.

ASP score range	Sample size	LR^d^ model	RF^e^ model	ANN^f^ model	RNN^g^ model	GRU^h^ model	LSTM^i^ model
1-1.5	1	0.266	0.825	0.358	0.586	0.452	0.726
1.5-2	13	0.269	0.338	0.235	0.234	0.229	0.428
2-2.5	23	0.833	0.252	0.438	0.350	0.270	0.243
2.5-3	15	0.612	0.381	0.442	0.331	0.400	0.365
3-3.5	6	0.512	0.641	0.445	0.746	0.288	0.503
3.5-4	1	0.571	1.149	0.985	1.278	0.467	1.163

^a^RMSE: root mean square error.

^b^ASP: antisocial personality.

^c^No samples featuring ASP scores of ≥4 were included in the testing set.

^d^LR: linear regression.

^e^RF: random forest.

^f^ANN: artificial neural network.

^g^RNN: recurrent neural network.

^h^GRU: gated recurrent unit.

^i^LSTM: long short-term memory.

An analysis of the model performance across the range of ASP scores revealed that although the LR model exhibited moderately good performance in the lower range (1-2, RMSE 0.266-0.269), its high RMSE values across the 2 to 3 range indicated that the model struggled in cases involving complex patterns. The RF model exhibited consistent performance in the middle range (1.5-3, RMSE 0.252-0.381) but was characterized by unstable extremes (3.5-4, RMSE 1.149). The ANN model maintained a relatively low RMSE (≤0.445) in the range of 1 to 3.5, suggesting consistent performance; however, in the range of 3.5 to 4, it exhibited an RMSE of 0.985, highlighting the limitations of these rare cases of high scores. The RNN model demonstrated strong performance in the intermediate range (1.5-3, RMSE 0.234-0.350); however, excessive error was observed in the extreme range in this context (3.5-4, RMSE 1.278). The GRU model excelled, exhibiting the lowest RMSE in the 1.5 to 2 range (0.229) alongside consistent performance across most ranges (≤0.467). In contrast, the LSTM model exhibited poor performance in the high range (3.5-4, RMSE 1.163), and its performance in the middle range was inferior to that of the GRU (eg, 2-2.5, RMSE 0.243 compared with 0.288 for the GRU), despite comparable results in the low range (1.5-2, RMSE 0.428 compared with 0.224 for the GRU).

Given its balanced performance and resilience in small samples, the GRU model was optimal for predicting ASP scores.

**Figure 5:**
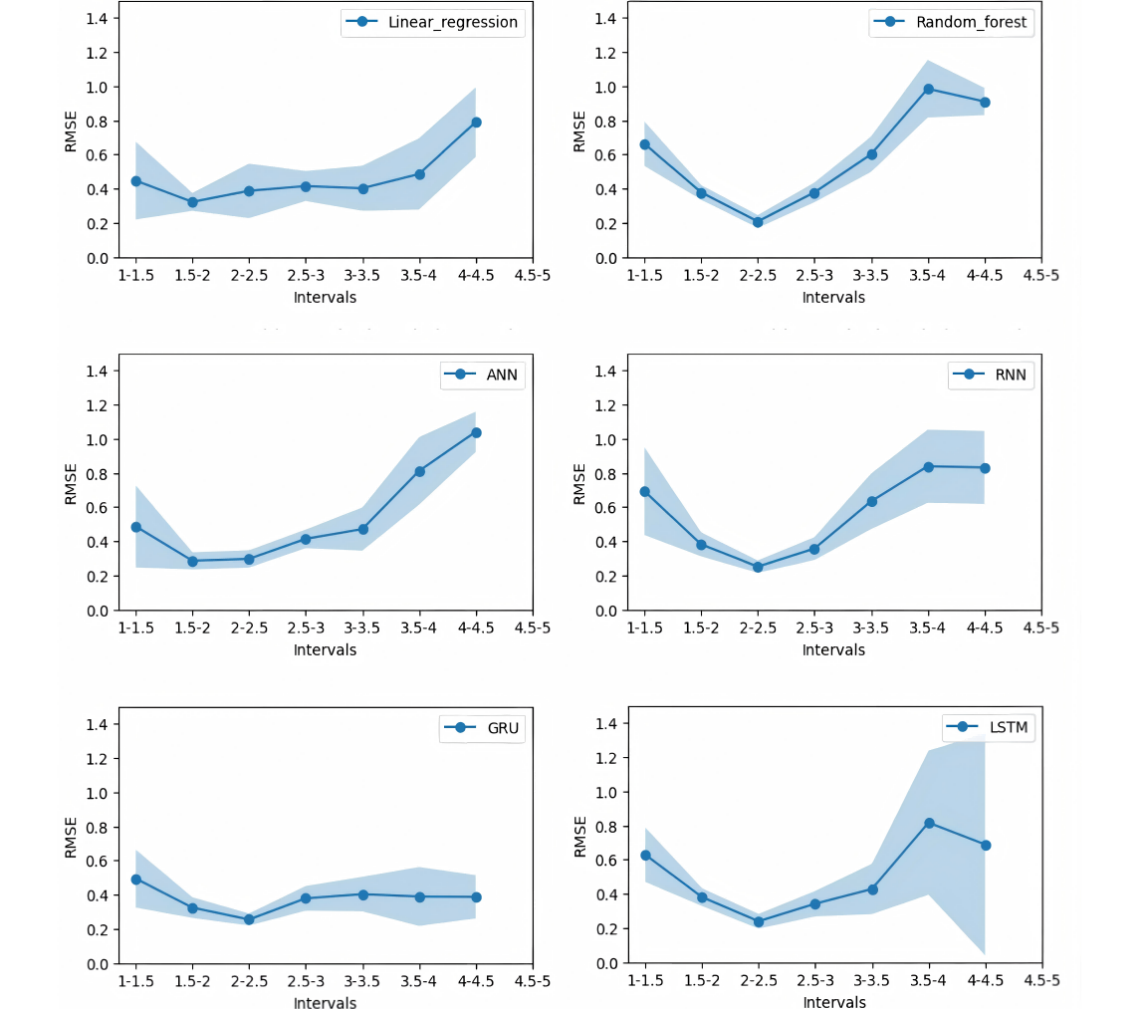
Root mean square error (RMSE) variations across 100 random testing set selections for different models (1-hot encoding). RMSE means are shown with SD bands. ANN: artificial neural network; GRU: gated recurrent unit; LSTM: long short-term memory; RNN: recurrent neural network.

This study used 100 different random seeds to facilitate random sampling of the test set, with the goal of examining the robustness of each model in greater detail. Across the 100 experiments, the study revealed changes in the RMSE values across various ASP score ranges. The results are presented in Figure 5.

#### Best Model

Notably, the tuning results varied depending on the data processing method used. When expert scoring was used, models often exhibited underfitting, whereas models that used 1-hot encoding frequently exhibited overfitting. However, both approaches can ultimately be adjusted to achieve satisfactory results through hyperparameter tuning.

A possible reason for this finding is that expert scoring relies on the team’s understanding and experience to assign values to each option. The expert scoring method, which involves limited feature dimensions (34×2), may not fully capture all behavioral patterns and dynamic changes, thereby reducing the model’s ability to capture comprehensive features and leading to underfitting. In contrast, 1-hot encoding increases the feature dimensions (34×5) and theoretically retains all the information contained in the original data. The higher-dimensional feature representation can capture more details but may also be impacted by noise and outliers, thus leading to overfitting.

Overall, the GRU model not only excelled in predictive robustness but also showed the strongest correlations between the predicted results and the actual observed values. Therefore, after all the indicators were balanced, we decided to use the GRU model as a predictive tool in the subsequent phases of this research.

Overall, the assessment model trained using the GRU model via 1-hot encoding data processing was selected as the final model because its correlation with the ASP questionnaire scores was higher than that of the scores obtained via the expert encoding process (*r*=0.792>0.742). Detailed results are referred to Table 10.

In addition, as part of this study, models were trained using each of the 7 behavioral traits of ASP as individual prediction labels. The results revealed that models trained on the total score exhibited performance comparable to that of models trained on individual traits, although the former achieved higher levels of precision. Specific details concerning this analysis are provided in Multimedia Appendix 4 for readers seeking further technical insights.

This study assessed the retest reliability of the ASP-ECD-G instrument using the intraclass correlation coefficients (ICCs). The results revealed a moderate to high level of reliability, as indicated by an ICC of 0.776 (95% CI 0.097-0.944, *df*=9; *P*=.02); these findings revealed consistent behavioral responses over a 1-month interval. This statistic exceeded the conventional threshold for acceptable reliability (ICC≥0.70), thus suggesting that measures of antisocial traits stabilized over time. The significant *P* value (*P*=.02<.05) confirmed that the observed retesting consistency was not the result of chance; these results thus indicated the instrument’s suitability for longitudinal assessment. Although the sample size was small, the robust ICC provides initial evidence to support the reliability of the gamified assessment; however, validation using a larger longitudinal sample is necessary.

**Table 10:** Descriptive statistics and correlation results of model predictions and questionnaire assessments.

Variables	Values, mean (SD)	Values, range	ASP^a^ questionnaire score	*P* value
ASP questionnaire score	2.303 (0.532)	4.357-1.107	—^b^	—
Expert scoring GRU^c^ model	2.383 (0.430)	4.172-1.578	0.742	<.001
1-hot encoding GRU model	2.317 (0.476)	4.264-1.479	0.792	<.001

^a^ASP: antisocial personality.

^b^Not applicable.

^c^GRU: gated recurrent unit.

### Analysis of Assessment Properties

#### Comparison of the Experience of Completing Gamified Assessments and Questionnaire Assessments

The results of the repeated-measures ANOVA are presented in Table 11. Notably, significant differences were evident in the experience dimensions between the gamified assessment results and the questionnaire assessment results (*F_147_*=25.522, 47.940, 5.581, 151.109, 14.259; *P*<.05).

A comparison concerning the pleasure dimension revealed that the pleasure associated with the gamified assessment was lower than that associated with the questionnaire assessment (*F*_147_=25.522; *P*<.001). This finding indicates that while participants might have experienced some degree of enjoyment during the gamified assessment, this approach may not have fully evoked the level of pleasure expected, possibly because of the conflict, contradictions, and negative features embedded in the gamified assessment scenarios, including the corresponding choices and outcomes (ie, receiving a “dismissal notice” from human resources). This misalignment with participants’ expectations could result in an unpleasant experience. However, this unpleasant experience suggests higher levels of engagement on the part of the participants, who were more likely to be influenced by the storyline, thus leading to higher levels of the immersion experience.

**Table 11:** Comparison of experience ratings between the gamified assessment and the questionnaire assessment (n=148, 2×2 mixed experimental design)^a^.

Variable and assessment type			Values, mean (SD)		Mean difference			*F* test (*df*)			*P* value			Partial η²	
**Pleasure**						–0.750			25.522 (147)			<.001			0.153
	ASP-ECD-G^b^	5.432 (2.158)													
	ASP^c^ questionnaire	6.182 (1.686)													
**Interest**						0.939			47.940 (147)			<.001			.254
	ASP-ECD-G	7.095 (1.643)													
	ASP questionnaire	6.155 (1.979)													
**Positive emotions**						0.392			5.581 (147)			.02			0.038
	ASP-ECD-G	6.507 (2.045)													
	ASP questionnaire	6.115 (2.062)													
**Negative emotions**						1.892			151.109 (147)			<.001			0.517
	ASP-ECD-G	4.365 (2.330)													
	ASP questionnaire	2.473 (1.809)													
**Immersion**						0.412			14.259 (147)			<.001			0.092
	ASP-ECD-G	7.628 (1.225)													
	ASP questionnaire	7.216 (1.720)													

^a^The gender, age, highest level of education, marital status, employment status, and position of the participants were included as control variables.

^b^ASP-ECD-G: Antisocial Personality Traits Evidence-Centered Design Gamified assessment tool.

^c^ASP: antisocial personality.

The interest generated by the gamified assessment was greater than that generated by the questionnaire assessment (*F*_147_=47.940; *P*<.001), thus indicating that the gamified assessment was relatively successful in providing an interesting and engaging experience. The introduction of gamified elements likely increased the interactivity and the rates of participation in the tasks, thereby increasing the participants’ interest and engagement.

The comparison of positive emotions also indicated that the gamified assessment evoked more positive emotions (*F*_147_=5.581; *P*=.02). This finding might be because of the reward mechanisms associated with the gamified assessment, which enhanced the participants’ sense of achievement and self-efficacy. This positive emotional experience may encourage the participants to engage honestly in the experiment to receive more positive feedback.

The results regarding negative emotions revealed that the scores pertaining to the gamified assessment significantly exceeded those pertaining to the questionnaire assessment (*F*_147_=151.109; *P*<.001). This finding is in line with the results concerning pleasure reported previously. The fact that the scores for both positive and negative emotions were higher in the gamified assessment suggests that the overall emotional state of the participants was more intense during the gamified assessment process, indicating higher levels of engagement that can, in turn, enhance immersion and authenticity.

The comparison of immersion experience revealed that the gamified assessment obtained a significantly higher value than the questionnaire assessment (*F*_147_=14.259; *P*<.001), thus indicating that the gamified assessment can capture the participants’ attention more effectively, thereby making them more focused on the task at hand. A cross-analysis of the gamified and questionnaire assessment scores is presented in Table 12. This study revealed that, among the participants, both assessment methods resulted in a high proportion of the participants who reported high satisfaction scores (ie, simultaneously choosing scores of 7, 8, or 9); furthermore, the immersion experience scores associated with the gamified assessment were consistently ≥4.

**Table 12:** Cross-tabulation of immersion experience scores between the gamified and questionnaire assessments^a^.

Immersion experience score			ASP^b^ questionnaire																			Total (n=148)
			1 (n=2)		2 (n=1)		3 (n=3)		4 (n=2)		5 (n=16)		6 (n=21)		7 (n=20)		8 (n=46)		9 (n=37)			
**ASP-ECD-G**																						
	4	0		0		0		0		0		2		0		0		0		2		
	5	0		0		0		0		5		2		0		0		0		7		
	6	1		1		2		1		4		5		3		1		0		18		
	7	0		0		0		0		5		9		12		5		1		32		
	8	1		0		1		0		2		1		4		28		10		47		
	9	0		0		0		1		0		2		1		12		26		42		

^a^No participants obtained immersion experience scores of 1 to 3 in the gamified assessment.

^b^ASP: antisocial personality.

^c^ASP-ECD-G: Antisocial Personality Traits Evidence-Centered Design Gamified assessment tool.

Subsequently, 2 participants who rated the immersion experience associated with the gamified assessment higher than that associated with the questionnaire assessment were randomly selected for follow-up interviews. These participants reported that the emphasis of the gamified assessment on interactivity and engagement enabled them to become more deeply absorbed in the experience. In contrast, the questionnaire assessment tended to represent participants’ retrospective evaluation of the overall experience, which could entail a slight disconnection from their current state. This feedback further indicates that gamified assessment tools can not only engage participants effectively but also sustain higher levels of immersion over longer periods than is possible through the use of questionnaires.

#### Examination of Resistance to Faking in the Gamified Assessment Questionnaires

The descriptive statistics pertaining to different game formats according to the presence or absence of participant payment incentives are presented in Table 13.

The results of the ANOVA, which are presented in Table 14, indicate significant differences in questionnaire scores between situations in which participant payment incentives were present and those in which they were absent (*F*_1_=5.740; *P*=.02). The participants who received payment incentives obtained significantly lower ASP scores than those who did not receive payment incentives. However, no significant differences were observed in the gamified assessment results (*F*_1_=0.138; *P*=.71).

Two possible explanations may account for this result. First, gamification itself can enhance the intrinsic appeal of the task, thus causing the participants to focus on the task itself rather than on potential external rewards. Second, gamification simulates a real game scenario, in which context the immersive experience that occurs during the response process increases the cost and difficulty of faking, thus making it challenging for the participants to manipulate their responses in line with their subjective goals. Regardless, the high resistance to faking that characterizes gamified assessment plays a crucial role in efforts to reflect the participants’ personal traits more authentically, especially in recruitment and selection contexts.

**Table 13:** Descriptive statistics for different game formats according to the presence or absence of participant payment incentives.

Assessment type and participant payment incentive			Values, mean (SD)		Sample size, n
**ASP-ECD-G^a^**					
	No	2.185 (0.430)		74	
	Yes	2.156 (0.382)		74	
**ASP^b^ questionnaire**					
	No	2.201 (0.420)		74	
	Yes	2.066 (0.373)		74	

^a^ASP-ECD-G: Antisocial Personality Traits Evidence-Centered Design Gamified assessment tool.

^b^ASP: antisocial personality.

**Table 14:** ANOVA results concerning resistance to response manipulation (n=148)^a^.

Source and dependent variable		Type III sum of squares	Mean square	*F* test (*df*)	*P* value	Partial η²
**Corrected model**						
	ASP^b^ questionnaire	2.073	0.296	1.915 (7)	.07	0.087
	ASP-ECD-G^c^	2.529	0.361	2.333 (7)	.03	0.104
**Intercept**						
	ASP questionnaire	4.090	4.090	26.455 (1)	<.001	0.159
	ASP-ECD-G	5.424	5.424	35.023 (1)	<.001	0.200
**Gender**						
	ASP questionnaire	0.450	0.450	2.912 (1)	.09	0.020
	ASP-ECD-G	0.175	0.175	1.128 (1)	.29	0.008
**Age (y)**						
	ASP questionnaire	0.006	0.006	0.039 (1)	.84	0.000
	ASP-ECD-G	0.021	0.021	0.136 (1)	.71	0.001
**Highest education**						
	ASP questionnaire	0.150	0.150	0.971 (1)	.33	0.007
	ASP-ECD-G	0.182	0.182	1.177 (1)	.28	0.008
**Marital status**						
	ASP questionnaire	0.007	0.007	0.048 (1)	.83	0
	ASP-ECD-G	1.087	1.087	7.018 (1)	.01	0.048
**Employment status**						
	ASP questionnaire	0.022	0.022	0.142 (1)	.71	0.001
	ASP-ECD-G	0.002	0.002	0.015 (1)	.90	0
**Position**						
	ASP questionnaire	0.642	0.642	4.155 (1)	.04	0.029
	ASP-ECD-G	0.337	0.337	2.176 (1)	.14	0.015
**Participant payment incentive**						
	ASP questionnaire	0.887	0.887	5.740 (1)	.02	0.039
	ASP-ECD-G	0.021	0.021	0.138 (1)	.71	0.001
**Error**						
	ASP questionnaire	21.644 (140)	0.155	—^d^	—	—
	ASP-ECD-G	21.680 (140)	0.155	—	—	—
**Total**						
	ASP questionnaire	697.505 (148)	—	—	—	—
	ASP-ECD-G	721.585 (148)	—	—	—	—
**Corrected total**						
	ASP questionnaire	23.717 (147)	—	—	—	—
	ASP-ECD-G	24.209 (147)	—	—	—	—

^a^Antisocial personality questionnaire: *R*²=0.087 (adjusted *R*²=0.042); Antisocial Personality Traits Evidence-Centered Design Gamified assessment tool: *R*²=0.104 (adjusted *R*²=0.060).

^b^ASP: antisocial personality.

^c^ASP-ECD-G: Antisocial Personality Traits Evidence-Centered Design Gamified assessment tool.

^d^Not applicable.

#### Analysis of Future Assessment Preferences

Despite the higher levels of negative emotions induced by the negative outcomes in the gamified assessment tool, at the end of the survey, approximately 79.1% of the participants indicated that they would prefer to participate in a gamified scenario assessment over a questionnaire assessment in future employee psychological assessments. This finding suggests that gamified assessment tools are inherently more appealing to participants and that they might provide them with more emotional experiences. Such enhanced emotional experience is likely the result of the content of the gamified assessment tool itself.

## Discussion

### Principal Findings

On the basis of the *DSM-5* criteria, 7 core antisocial traits were operationalized into 24 observable workplace behaviors based on semistructured interviews conducted with 9 industry professionals (Table 1). These behaviors were integrated into a text-based gamified assessment that included 10 subscenarios and 34 interactive questions with a logic rooted in logical jumps (eg, question jumps and progressive paths), thereby ensuring alignment between clinical constructs and simulated workplace decisions (Multimedia Appendix 1). Content validity was confirmed based on an expert review; in this context, 100% correspondence was observed between game behaviors and *DSM-5* traits.

Six machine learning models were compared using PID-5-SF scores as criteria (n=286). The GRU model exhibited optimal performance, achieving a criterion correlation of *r*=0.850 and a test set prediction error of RMSE 0.273 (Table 6). An analysis conducted across different ASP score ranges revealed the stability of the GRU in the midscore range (1.5-3, RMSE ≤0.377) and resistance to extreme value fluctuations (3.5-4, RMSE 0.854), whereas the LR and LSTM models exhibited greater errors in complex or sparse score intervals (Table 7). A supplementary reliability study conducted with 10 participants yielded a moderate-to-strong ICC (0.776, 95% CI 0.097-0.944, *df*=9, *P*=.02), thus highlighting the temporal stability of scores over a 1-month interval (refer to the Results section, Best Model subsection).

A 2×2 mixed experimental design (n=148) revealed significant differences in immersion experience between gamified assessment (mean 7.628) and questionnaire assessment (mean 7.216; *F*_147_=14.259; *P*<.001; Table 11). External incentives (ie, increased participation payments) induced response bias in the questionnaires, such that incentivized participants reported lower ASP scores (mean 2.066 vs 2.201; *F*_1_=5.740; *P*=.02), whereas gamified assessment scores remained unaffected (*F*_1_=0.138; *P*=.71; Table 14), thus indicating that the narrative effectively neutralized strategic self-presentation (Table 14). The gamified assessment also elicited stronger negative emotions (mean 4.365 vs 2.473; *F*_147_=151.109; *P*<.001) and higher levels of interest (mean 7.095 vs 6.155; *F*_147_=47.940; *P*<.001) in line with its immersive design. The variance decomposition revealed that immersion experience served as an effective antibias component (*F*_147_=14.259; *P*<.001, η²=0.092).

The future assessment preference data indicated that 79.1% (117/148) of the participants preferred the gamified assessment over questionnaires, despite the stronger negative emotions induced by the gamified assessment, thus reflecting the acceptability of this approach in organizational settings (Table 12).

This study reports the construction of a replicable ECD framework for gamified psychological assessment, which can be extended beyond the assessment of antisocial traits to encompass broader personality constructs. The framework involves six iterative phases: (1) theoretical and needs analysis, which aligns target constructs (eg, *DSM-5* criteria) with the empirical literature and stakeholder requirements; (2) competency model construction, which involves translating abstract traits into hierarchical, observable behavioral indicators; (3) evidence model development, which entails linking behavioral manifestations with trait dimensions based on expert validation; (4) game ontology design, which focuses on embedding assessment tasks within immersive narratives via dynamic decision paths; (5) technical implementation, which requires integrating interactive interfaces with mechanisms for the capture of real-time data; and (6) model training and validation, which highlights the use of machine learning algorithms to transform behavioral patterns into reliable trait scores that can be validated by reference to established instruments.

Key innovations include the integration of realistic scenarios to reduce response bias; the use of adaptive technology solutions (eg, dynamic jump logic and 1-shot coding) to capture nuanced behaviors; and multidimensional validation through statistical and qualitative methods. This framework, which prioritizes consistency among theoretical constructs, observable behaviors, and technical design, provides a systematic approach to the development of gamification tools that contribute to the broader aim of capturing realistic psychological traits.

### Limitations

Although significant progress has been made in the development and validation of the gamified assessment tool, several limitations warrant further investigation.

First, the validation experiments reported in this study relied on a limited sample. Future researchers should investigate more diverse samples, including those from different cultural backgrounds, types of organizations, and employee levels, with the aim of enhancing and validating the generalizability of this tool.

Second, although the tool resists falsification, the underlying psychological mechanisms remain unclear. Future researchers should explore how different gamified elements influence behavior and decision-making and design experiments aimed at elucidating these mechanisms.

While the initial retest analysis indicated acceptable reliability (ICC=0.776), the small sample size limited confidence in longitudinal stability. Future researchers could validate the reliability of this approach based on larger samples and longer time intervals (eg, 3-6 months), thereby enabling them to explore the validity of the assessment of the ASP-ECD-G in further detail.

### Comparison With Previous Work

Unlike traditional tools, such as scales and SJTs, which assume item independence and 1-dimensionality, the ASP-ECD-G uses interconnected items within a simulation, thereby reflecting a multidimensional understanding of antisocial traits. This innovative approach can facilitate a more comprehensive and accurate assessment of personality traits. Recent efforts to gamify personality assessment, such as the framework for integrity testing by Cui et al [29], have emphasized scenario-based design but have lacked systematic integration with clinical diagnostic criteria, such as the *DSM-5*. In contrast, this study explicitly anchors the assessment ontology in *DSM-5* antisocial traits, thereby ensuring content validity and connecting clinical theory with organizational contexts. This ECD approach is superior to ad hoc game development, as it creates a replicable pipeline leading from trait definition to behavioral evidence collection—a step often overlooked by previous gamified tools [30].

### Future Work

Regarding technology, this study used the GRU model for data processing. As artificial intelligence and machine learning technologies continue to develop, future researchers should explore advanced prediction algorithms and expand the sample of participants to improve data analysis capabilities and prediction accuracy. In addition, the current questionnaire-based format of this tool is relatively monotonous. The integration of multimodal data (eg, physiological responses or interaction timing) and the development of software that includes situational images and character avatars—with the goal of deepening behavioral insights—are expected to create a more immersive and engaging assessment experience, thereby improving user engagement and ecological validity.

As a foundational step in the process of developing a gamified assessment for ECD, addressing limitations remains critical. Issues that must still be addressed include sample diversification across groups with different cultural backgrounds, occupations, ages, and levels of education, with the aim of determining whether cross-context and cross-domain applications continue to exhibit utility in these contexts. Future work should also explore the temporal boundaries of the validity of relevant measurements by extending the retesting intervals (eg, 6-12 months). Technical improvements such as visual scenario design and adaptive algorithm improvements may further enhance immersion and measurement accuracy. This study highlights the potential of behavioral assessments to complement traditional self-report methods by anchoring gamification tools in systematic frameworks, such as the ECD, and by emphasizing rigorous validation. This approach provides new ways of understanding negative traits in a more nuanced and reliable manner.

### Conclusions

The development and validation of the ASP-ECD-G address, in part, the critical limitations of traditional self-report tools used to assess complex antisocial traits by providing a behavior-based, immersive alternative. The ASP-ECD-G exhibits robust content validity (100% alignment with *DSM-5* traits), excellent predictive accuracy (GRU model criterion correlation *r*=0.792), and moderate to strong test-retest reliability (ICC=0.776; *P*=.02). It also exhibits resistance to response manipulation, as incentivized participants in this research were associated with altered questionnaire scores but not gamified assessment results (*P*=.71), thus supporting the stability of this tool over time and identifying it as a reliable instrument for capturing antisocial tendencies in simulated workplace scenarios.

Unlike self-report tools, which are susceptible to social desirability bias, the gamified approach elicits authentic responses through immersive decision-making, as evidenced by significantly higher immersion scores (mean 7.628 vs 7.216; *P*<.001) and consistent performance under conditions involving incentives. This mechanism, which is supported by flow theory, is correlated with reduced response distortion. These findings highlight the potential of ECD-based gamified assessments to improve the accuracy of personality measurement in high-stakes contexts, such as recruitment, where traditional methods often fail to detect subtle antisocial behaviors.

Ultimately, the ASP-ECD-G offers a theoretically sound and empirically validated framework, thereby contributing to both psychological research on negative personality traits and organizational practices aimed at mitigating the associated risks. Although this tool is not definitive or final, its contribution lies in the fact that it offers a replicable methodology and preliminary evidence for gamified assessment as a credible alternative in the context of trait measurement, thus calling for continued innovation to unlock its full potential for real-world applications.

## Appendix

Multimedia Appendix 1 Summary of interview results and interview questionnaire.

Multimedia Appendix 2 The presentation of the Antisocial Personality Traits Based on Evidence-Centered Design Gamified assessment tool.

Multimedia Appendix 3 Assessment items pertaining to pleasure, fun, positive emotions, negative emotions, and immersion experience, as well as the Personality Inventory for DSM-5–Short Form items used to measure antisocial personality traits.

Multimedia Appendix 4 Model prediction results for different behavioral traits.

Multimedia Appendix 5 Validation of the multidimensional measurement used in gamified assessment tools.

Multimedia Appendix 6 CONSORT-eHEALTH checklist (V 1.6.1).

## Abbreviations

ANN: artificial neural network
ASP: antisocial personality
ASP-ECD-G: Antisocial Personality Traits Evidence-Centered Design Gamified assessment tool
DSM-5: Diagnostic and Statistical Manual of Mental Disorders, Fifth Edition
ECD: evidence-centered design
GRU: gated recurrent unit
ICC: intraclass correlation coefficient
LR: linear regression
LSTM: long short-term memory
MAE: mean absolute error
PID-5-SF: Personality Inventory for DSM-5 Short Form
RF: random forest
RMSE: root mean square error
RNN: recurrent neural network
SJT: situational judgment test

## Data Availability

The datasets generated or analyzed during this study are available from the corresponding author on reasonable request.

## References

[1] Hogan R, Hogan J (2008). Assessing leadership: a view from the dark side. Int J Sel Assess.

[2] Fossati A, Barratt ES, Carretta I, Leonardi B, Grazioli F, Maffei C (2004). Predicting borderline and antisocial personality disorder features in nonclinical subjects using measures of impulsivity and aggressiveness. Psychiatry Res.

[3] Robinson SL, O'Leary-Kelly AM (1998). Monkey see, monkey do: the influence of work groups on the antisocial behavior of employees. Acad Manage J.

[4] Lilienfeld SO (1994). Conceptual problems in the assessment of psychopathy. Clin Psychol Rev.

[5] Maples JL, Carter NT, Few LR, Crego C, Gore WL, Samuel DB, Williamson RL, Lynam DR, Widiger TA, Markon KE, Krueger RF, Miller JD (2015). Testing whether the DSM-5 personality disorder trait model can be measured with a reduced set of items: an item response theory investigation of the Personality Inventory for DSM-5. Psychol Assess.

[6] Baumert A, Schlösser T, Schmitt M (2014). Economic games. Eur J Psychol Assess.

[7] Ployhart RE, Ehrhart MG (2003). Be careful what you ask for: effects of response instructions on the construct validity and reliability of situational judgment tests. Int J Sel Assess.

[8] Hare RD (1991). Psychopathy checklist—revised. APA PsycTests.

[9] Reyna VF, Farley F (2006). Risk and rationality in adolescent decision making: implications for theory, practice, and public policy. Psychol Sci Public Interest.

[10] Hare RD (1980). A research scale for the assessment of psychopathy in criminal populations. Pers Individ Dif.

[11] Coccaro EF, Lee R, McCloskey MS (2014). Relationship between psychopathy, aggression, anger, impulsivity, and intermittent explosive disorder. Aggress Behav.

[12] van Nimwegen C, van Oostendorp H, Modderman J, Bas M (2011). A test case for GameDNA: conceptualizing a serious game to measure personality traits. Proceedings of the 16th International Conference on Computer Games.

[13] Lievens F, Sackett PR (2012). The validity of interpersonal skills assessment via situational judgment tests for predicting academic success and job performance. J Appl Psychol.

[14] Deterding S, Dixon D, Khaled R, Nacke L (2011). From game design elements to gamefulness: defining "gamification". Proceedings of the 15th International Academic MindTrek Conference: Envisioning Future Media Environments.

[15] DiCerbo KE (2013). Game-based assessment of persistence. Educ Technol Soc.

[16] Teng CI (2008). Personality differences between online game players and nonplayers in a student sample. Cyberpsychol Behav.

[17] Mislevy RJ, Steinberg LS, Almond RG (2003). Rejoinder to commentaries for "on the structure of educational assessments". Meas Interdiscip Res Perspect.

[18] Zieky MJ (2014). An introduction to the use of evidence-centered design in test development. Psicol Educ.

[19] Shute VJ, Tobias S, Fletcher JD (2011). Stealth assessment in computer-based games to support learning. Computer Games and Instruction.

[20] Singh BK, Katiyar M, Gupta S, Ganpatrao NG (2021). A survey on: personality prediction from multimedia through machine learning. Proceedings of the 5th International Conference on Computing Methodologies and Communication.

[21] Ryan RM, Deci EL (2000). Self-determination theory and the facilitation of intrinsic motivation, social development, and well-being. Am Psychol.

[22] Csikszent M (1991). Flow: The Psychology of Optimal Experience.

[23] Lee SJ, Jeong EJ, Kim DJ, Kong J (2023). The influence of psychological needs and motivation on game cheating: insights from self-determination theory. Front Psychol.

[24] Liu L, Chen X, Szolnoki A (2023). Coevolutionary dynamics via adaptive feedback in collective-risk social dilemma game. Elife.

[25] First MB (2013). Diagnostic and statistical manual of mental disorders, 5th edition, and clinical utility. J Nerv Ment Dis.

[26] McKinley S, Patrick C, Verona E (2018). Antisocial personality disorder: neurophysiological mechanisms and distinct subtypes. Curr Behav Neurosci Rep.

[27] Bandura A (1999). Moral disengagement in the perpetration of inhumanities. Pers Soc Psychol Rev.

[28] Aquino K, Douglas S (2003). Identity threat and antisocial behavior in organizations: the moderating effects of individual differences, aggressive modeling, and hierarchical status. Organ Behav Hum Decis Process.

[29] Cui Y, Chu MW, Chen F (2019). Analyzing student process data in game-based assessments with Bayesian knowledge tracing and dynamic Bayesian networks. J Educ Data Min.

[30] Wouters P, van Nimwegen C, van Oostendorp H, van der Spek ED (2013). A meta-analysis of the cognitive and motivational effects of serious games. J Educ Psychol.

