# Supplementary material for: A Gamified Assessment Tool for Antisocial Personality Traits (Antisocial Personality Traits Evidence-Centered Design Gamified): Randomized Controlled Trial
Source: JMIR Serious Games. 2025 Aug 25;13:e70453. doi: 10.2196/70453 (PMC12417903; doi:10.2196/70453)
Supplement: Multimedia Appendix 1 [file games_v13i1e70453_app1.docx]

### Appendix 1: Summary of Interview Results & **Interview Questionnaire**

| **No.** | **Machiavellism** | **Callousness** | **Deceitfulness** | **Hostility** | **Risk-taking** | **Impulsivity** | **Irresponsibility** |
| --- | --- | --- | --- | --- | --- | --- | --- |
| 1 | When collaborating with other companies, some colleagues' first reaction is to protect themselves and shift blame to others |  | Employees exploit loopholes during hotel reimbursement to seek personal gain and are dismissed or resign when discovered by the company | Engineers engage in disputes with clients without consulting the project manager | Some colleagues download company-related data to their personal computers due to a fluke mindset | Engineers argue with clients without project manager consultation | Some engineers display a negative attitude towards work and refuse to execute tasks on unreasonable grounds, showing a burnout mentality |
| 2 |  | Sales director embezzles, and sales manager injects counterfeit goods into company channels |  | Manager impulsively proposes ideas that contradict company operations repeatedly | Manager is impatient with uncooperative colleagues, showing urgency and anger | Two managers often fail to complete tasks on time due to their habitual slack |  |
| 3 | Programmer allows his wife to face the company's questions while he remains silent | Programmer plagiarizes code from the internet and claims it as his own work | Programmer, dissatisfied with the company's decision on his misconduct, initiates arbitration with his wife against the company | Colleague disputes intensely with the CEO over task collaboration, disregarding the risk of termination | Colleague gets angry when others do not cooperate, yelling loudly during outbursts | Colleague demonstrates poor communication skills and fails to complete tasks as required |  |
| 4 | Veteran employees assign meaningless tasks to new hires to highlight their leadership abilities |  | Employee conceals pregnancy and uses fake GPS location to clock in from home |  | Employee hides pregnancy by clocking in from home using fake GPS, minimizing company contact | Department leader has inappropriate relations with female colleagues and posts improper photos on social media |  |
| 5 | Leader hides his transfer news from subordinates, possibly out of self-interest |  | Employee accepts bribes, deceives the company and drivers |  | Employee anonymously posts complaints about upper management on the intranet, risking job security | Leader loses temper over minor errors, displaying impulsive behavior | Employee neglects duties and fails to complete survey work on time, showing irresponsibility |
| 6 | Marketing staff privately contact clients for higher commissions, possibly out of self-interest |  | Editor reuses previous company's articles without reworking, deceiving the current company | Colleagues fight over clients, showing hostility | Colleague outsources project modification without acknowledging mistakes after disapproval from leadership | Leader loses temper due to miscommunication with subordinates, showing impulsiveness | Interns work late to complete projects due to high workload |
| 7 |  | Factory compromises product quality during production to reduce costs, submitting defective goods to clients |  | Factory's decision on quality control, balancing costs and benefits, avoids investing in precision instruments |  | Employee's mistakes, such as mislabeling, and factory's negligence on quality issues |  |
| 8 | Colleague violates company rules, frequently late, and makes excuses to evade responsibility | Colleague fails to honor commitments, agreeing verbally but not executing assigned tasks | Leader's poor communication leads to impulsive reactions from subordinates |  | Colleague shows reluctance to correct mistakes when pointed out by leaders | Some colleagues display a negative attitude towards mistakes, unwilling to correct them |  |
| 9 | Boss retains employees with promises of benefits and training opportunities but does not fulfill them later | Falsifies data to secure projects | Takes interns' plans |  | Boss uses promises of benefits and training opportunities to retain employees but does not fulfill them later | Takes interns' plans and resources | Intense competition over projects, including disputes, strong dissatisfaction, and public criticism |
