# Supplementary figures and images for: A Gamified Assessment Tool for Antisocial Personality Traits (Antisocial Personality Traits Evidence-Centered Design Gamified): Randomized Controlled Trial
Source: JMIR Serious Games. 2025 Aug 25;13:e70453. doi: 10.2196/70453 (PMC12417903; doi:10.2196/70453)

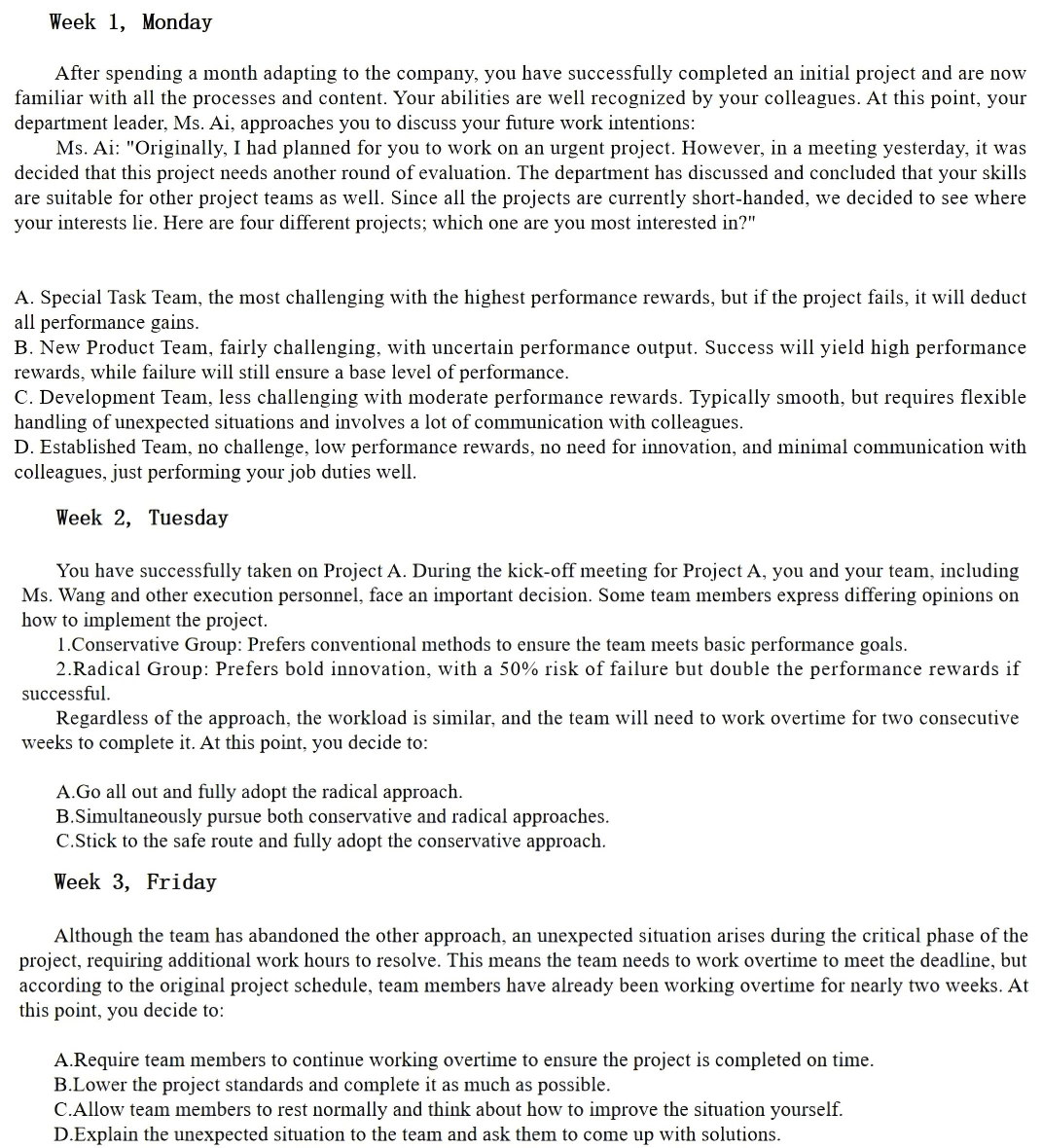

Supplement: Multimedia Appendix 2 [file games_v13i1e70453_app2.png]
