# Supplementary material for: A Gamified Assessment Tool for Antisocial Personality Traits (Antisocial Personality Traits Evidence-Centered Design Gamified): Randomized Controlled Trial
Source: JMIR Serious Games. 2025 Aug 25;13:e70453. doi: 10.2196/70453 (PMC12417903; doi:10.2196/70453)
Supplement: Multimedia Appendix 3 [file games_v13i1e70453_app3.docx]

### Appendix 2: assessment Items for Pleasure, Fun, Positive Emotions, Negative Emotions, and Immersion Experience & **DSM-5 Simplified Personality Inventory (PID-5-SF) Items for Measuring Antisocial Personality Traits**

**Pleasure:** While answering the gamified assessment items, how pleasurable were your emotions? "Not pleasurable" indicates feelings of discomfort, anger, or anxiety, whereas "pleasurable" indicates feelings of comfort and happiness. Higher values indicate higher levels of pleasure. (1 very unpleasurable - 9 very pleasurable)

**Fun:** How enjoyable did you find the gamified assessment items? If "not fun," it means you felt bored or disliked it; if "fun," it means you found it interesting and enjoyable. Higher values indicate a higher level of fun. (1 not at all - 9 extremely)

**Positive Emotions:** Rate the intensity of positive emotions you are experiencing at this moment. Higher values indicate a higher intensity of positive emotions. (1 not at all - 9 extremely)

**Negative Emotions:** Rate the intensity of negative emotions you are experiencing at this moment. Higher values indicate a higher intensity of negative emotions. (1 not at all - 9 extremely)

**Immersion Experience:** I feel as if I am really entering the world of the problems and am completely absorbed by these items. (1 not at all - 9 strongly agree)

**DSM-5 Simplified Personality Inventory (PID-5-SF) Items for Measuring Antisocial Personality Traits**

| **No.** | **Behavioral Trait** | **Item** |
| --- | --- | --- |
| 1 | Machiavellism | I am good at getting people to do what I want them to do. |
| 2 | Machiavellism | I am good at flattering colleagues to get the results I want. |
| 3 | Machiavellism | I am skilled at manipulating others. |
| 4 | Machiavellism | I easily get resources from others. |
| 5 | Callousness | I do not care whether my actions affect my colleagues. |
| 6 | Callousness | I do not care if my actions hurt others. |
| 7 | Callousness | If my words or actions hurt my colleagues, I do not think it is a big deal. |
| 8 | Callousness | I am indifferent to others' problems. |
| 9 | Deceitfulness | I often make up things to help achieve my goals. |
| 10 | Deceitfulness | If deceiving others could help me get a promotion or raise, I would be happy to do it. |
| 11 | Deceitfulness | I frequently get others to complete my tasks. |
| 12 | Deceitfulness | If it benefits me, I will distort the facts. |
| 13 | Hostility | I get angry easily at work. |
| 14 | Hostility | I have a bad temper at work. |
| 15 | Hostility | Work-related matters easily anger me. |
| 16 | Hostility | I am usually hostile towards certain colleagues. |
| 17 | Risk-taking | I do not stop myself from engaging in risky activities. |
| 18 | Risk-taking | I have done many things that others consider dangerous. |
| 19 | Risk-taking | I like taking risks. |
| 20 | Risk-taking | I do whatever I want, regardless of how unsafe it is. |
| 21 | Impulsivity | I feel my actions are entirely impulsive. |
| 22 | Impulsivity | I usually act on impulse without considering the consequences. |
| 23 | Impulsivity | Even though I know I can do better, I often stop after achieving a mediocre outcome. |
| 24 | Impulsivity | I always act impulsively. |
| 25 | Irresponsibility | I often neglect details in my work. |
| 26 | Irresponsibility | I do not necessarily fulfill the tasks I promise. |
| 27 | Irresponsibility | I frequently forget to pay bills or clear debts. |
| 28 | Irresponsibility | If I make a mistake, it is best to avoid taking responsibility. |
