# Supplementary material for: A Gamified Assessment Tool for Antisocial Personality Traits (Antisocial Personality Traits Evidence-Centered Design Gamified): Randomized Controlled Trial
Source: JMIR Serious Games. 2025 Aug 25;13:e70453. doi: 10.2196/70453 (PMC12417903; doi:10.2196/70453)
Supplement: Multimedia Appendix 4 [file games_v13i1e70453_app4.docx]

### Appendix 5: Model Prediction Results for Different Behavioral Traits

This section discusses the modeling and prediction analysis for the seven behavioral traits of antisocial personality as individual prediction targets. The models used the optimal GRU model settings obtained from the training results of Study 2, with adjustments only to the learning rate. The input remained unchanged, using one-hot encoding, and the output was replaced with the scores of the seven behavioral traits measured by the PID-5-SF questionnaire. The specific prediction model results for each behavioral trait are shown in Table 16.

1. Model Prediction Results for Individual Antisocial Traits (Seven DSM-5 Traits, n = 286)

| **Data Set** | **Evaluation Metric** | **Machiavellism** | **Callousness** | **Deceitfulness** | **Hostility** | **Risk-taking** | **Impulsivity** | **Irresponsibility** |
| --- | --- | --- | --- | --- | --- | --- | --- | --- |
| Training Set | RMSE | .641 | .623 | .471 | .749 | .673 | .695 | .477 |
|  | MAE | .531 | .48 | .358 | .626 | .542 | .538 | .386 |
|  | r | .250 | .505 | .807 | .352 | .442 | .348 | .725 |
| Testing Set | RMSE | .703 | .633 | .519 | .730 | .660 | .698 | .541 |
|  | MAE | .587 | .469 | .371 | .604 | .579 | .581 | .416 |
|  | r | .315 | .496 | .763 | .333 | .303 | .274 | .483 |
| Note: r represents the correlation between the predicted results of the optimal model and the reference results. | | | | | | | | |

The model training results indicate that there is no significant overfitting or underfitting issues for any of the models. Among the prediction results on the testing set, "Deceitfulness" has the lowest RMSE and MAE values and the highest correlation with the reference results, indicating the best overall prediction accuracy. In contrast, "Hostility," "Impulsivity," and "Risk-taking" exhibit higher RMSE and MAE values and lower correlations with the reference results, suggesting lower prediction accuracy for these traits.

Several factors may explain these differences. First, deceitfulness may have more consistent behavioral patterns, making it easier for the model to predict. Hostility, impulsivity, and risk-taking are likely more influenced by situational factors, resulting in greater variability and lower prediction accuracy. Second, the PID-5-SF questionnaire may more accurately measure deceitfulness than other traits, impacting the model's performance. Third, the GRU model may be more sensitive to deceitfulness-related data, while dynamic behaviors like hostility and impulsivity may require additional contextual data for better prediction. Lastly, sample characteristics could influence model performance, with more consistent patterns of deceitfulness in the sample leading to better predictions. Future research should consider incorporating additional data sources, refining measurement tools, and exploring alternative modeling approaches to improve prediction accuracy for complex traits, thereby enhancing the effectiveness of the gamified assessment tool.

The study set up 100 different random seeds for the random selection of the testing set and observed the RMSE value changes across the 100 experiments for different behavioral trait scores. The results are shown in Figure 5.


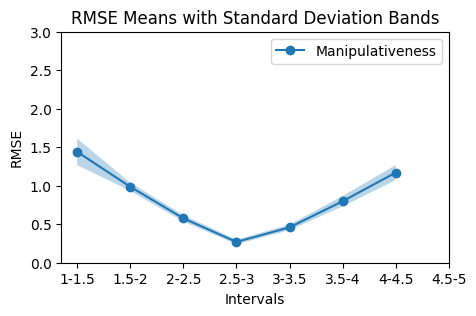

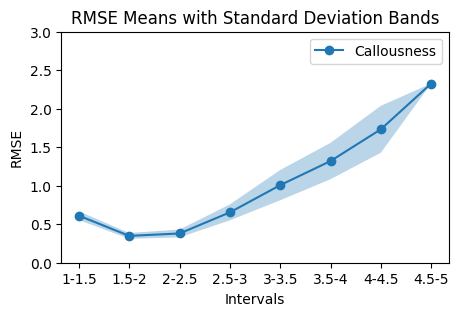

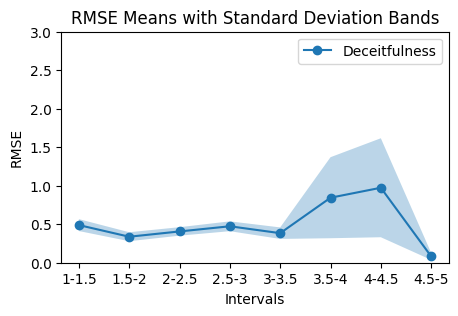

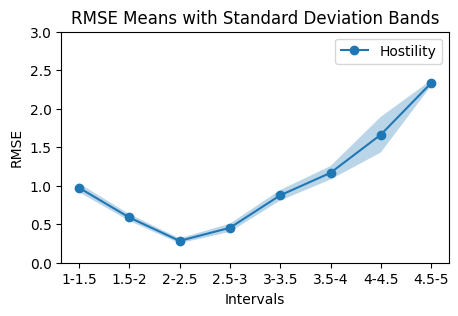

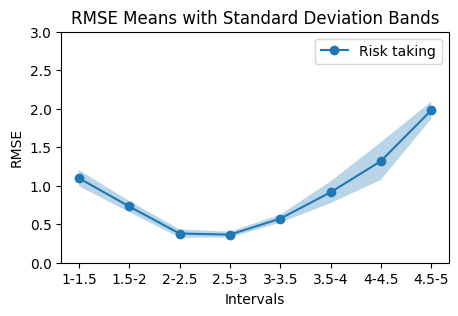

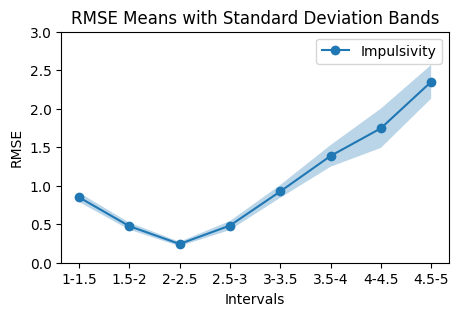

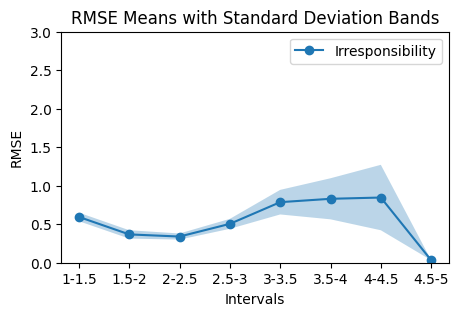


Note: The line represents the average of 100 experiments, and the light blue range represents the mean ± 1 standard deviation.

1. RMSE Variations in the Testing Set for Different Behavioral Trait Prediction Models Across 100 Random Selections

Overall, the models' prediction performance for individual behavioral traits is lower compared to antisocial personality as a whole. For scores above 3, the RMSE values of all models exceed 0.5, with significant fluctuations observed in the prediction performance for "Deceitfulness" and "Irresponsibility."

1. Descriptive Statistics and Correlations Between Model Predictions and Questionnaire assessments

| **Pairing** | **assessment Form** | **Mean** | **Cases** | **Std. Deviation** | **Correlation** | **t** |
| --- | --- | --- | --- | --- | --- | --- |
| Pair 1 | Model 1 | 2.227 | 58 | .375 | .586(*P* < .001) | -.799 |
|  | Reference Result | 2.267 | 58 | .456 |  |  |
| Pair 2 | Model 2 | 2.277 | 58 | .218 | .513(*P* < .001) | .181 |
|  | Reference Result | 2.267 | 58 | .456 |  |  |
| Note: Model 1 represents the antisocial personality score prediction using the optimal model from Study 2; Model 2 represents the antisocial personality score prediction obtained by averaging the predictions of the seven behavioral trait models; the reference result is the score measured by the ASP questionnaire. | | | | | | |

The comparative analysis of the prediction models and questionnaire assessments is shown in Table 17. Paired sample t-test results indicate no significant difference between the prediction scores and the reference results for both Model 1 (t = -.799, *P* > .05) and Model 2 (t = .181, *P* > .05). Both Model 1 (r = .586, *P* < .01) and Model 2 (r = .513, *P* < .01) have significant correlations with the reference results, indicating that both models can effectively predict antisocial personality scores. However, Model 1 shows a slightly higher correlation, suggesting a better fit with the questionnaire data.

When using ASP-ECD-G for antisocial personality assessment, the prediction effectiveness of directly training a model based on the total score is comparable to averaging the predictions of individual behavioral trait models. Notably, the direct total score model performs better in terms of prediction accuracy. This may be due to the inherent interaction between antisocial personality traits, which are likely to manifest in a holistic manner in individual behavior rather than in isolation. This means that a direct total score model may better capture the internal relationships and interaction effects between these traits, providing more accurate predictions.

From a statistical perspective, participants' behavioral responses in gamified assessments may be influenced by multiple antisocial personality traits simultaneously, leading to complex interactions between multidimensional features. This requires models to learn not only independent representations of traits but also their joint representations. The total score model may benefit from simplified assumptions and fewer parameter estimations, helping to reduce model complexity and overfitting risk. Conversely, combining predictions from multiple behavioral trait models might introduce noise if individual trait assessment errors are significant, thereby reducing overall prediction accuracy.
