# Supplementary material for: A Gamified Assessment Tool for Antisocial Personality Traits (Antisocial Personality Traits Evidence-Centered Design Gamified): Randomized Controlled Trial
Source: JMIR Serious Games. 2025 Aug 25;13:e70453. doi: 10.2196/70453 (PMC12417903; doi:10.2196/70453)
Supplement: Multimedia Appendix 5 [file games_v13i1e70453_app5.docx]

### Appendix 4: Validation of Multidimensional Measurement in Gamified Assessment Tools

Traditional assessment forms, such as scales, Situational Judgment Tests (SJT), involve questions and options where respondents select the option that best reflects their situation or preference. These methods assume independence and one-dimensionality, meaning responses to different questions are independent, and each question measures a single trait or ability. Scales primarily focus on attitudes or behavioral tendencies without scenarios, whereas SJT incorporate scenarios into their design. Scoring for scales typically uses Likert scales; SJT options (usually 3-5) are scored by assigning predetermined values and summing or averaging scores.

The validation of our ASP-ECD-G gamified assessment tool requires different methods from traditional questionnaires due to three main differences:

1. Data Type: Traditional questionnaires generate ordinal data through self-reports on uniform scales. In contrast, ASP-ECD-G collects nominal data through participant decisions in simulated scenarios, with varying options (2-5) and sequentially related items.
2. Item Independence: Traditional validity testing methods, based on Classical Test Theory (CTT) or Item Response Theory (IRT), assume item independence. ASP-ECD-G, however, includes interconnected items, where responses to subsequent items are influenced by previous answers within a simulated scenario.
3. Dimensionality: Traditional assessments assume one-dimensionality, with each item measuring a single dimension. In ASP-ECD-G, each item reflects a behavioral decision influenced by multiple dimensions, invalidating the one-dimensionality assumption.

Therefore, validating gamified assessment tools like ASP-ECD-G requires considering data type, item interrelation, and multidimensionality, necessitating complex statistical methods. Although modeling with IRT is theoretically challenging, we attempted it using the Nominal Response Model (NRM) with the mirt package in R. Exploratory analysis on 34 items is presented in Table 15.

1. Exploratory Modeling Analysis Results of NRM

| **Dimension** | **1** | **2** | **3** | **4** | **5** | **6** | **7** |
| --- | --- | --- | --- | --- | --- | --- | --- |
| Iteration | 53 | 86 | 78 | 92 | 78 | 246 | 16 |
| Log-Lik | -5673.948 | -5379.665 | -5237.799 | -5149.281 | -5191.651 | -6640.377 | -6640.371 |
| Max-Change | 0 | 0 | 0 | 0 | 0 | 0 | 0 |
| AIC | 11715.90 | 11193.33 | 10973.60 | 10858.56 | 11003.30 | 13958.75 | 14014.74 |
| BIC | 12336.95 | 11925.77 | 11814.04 | 11803.64 | 12049.64 | 15102.97 | 15253.47 |
| logLik | -5673.948 | -5379.665 | -5237.799 | -5149.281 | -5191.651 | -6640.377 | -6640.371 |
|  | —— | 588.566 | 283.732 | 177.036 | -84.739 | -2897.453 | .014 |
| df | —— | 33 | 32 | 31 | 30 | 29 | 28 |
| p | —— | 0 | 0 | 0 | —— | —— | 1 |
| M2 | 1459.341 | 1113.683 | 886.196 | 536.520 | 904.890 | 1551.799 | 1486.637 |
| df | 411 | 378 | 346 | 315 | 285 | 256 | 228 |
| p | 0 | 0 | 0 | 0 | 0 | 0 | 0 |
| RMSEA | .109 | .095 | .085 | .057 | .101 | .153 | .160 |
| RMSEA_5 | .103 | .088 | .078 | .049 | .093 | .146 | .152 |
| RMSEA_95 | .115 | .101 | .092 | .065 | .108 | .160 | .168 |
| TLI | .689 | .762 | .809 | .914 | .734 | .382 | .326 |
| CFI | .745 | .821 | .869 | .946 | .850 | .685 | .694 |

The IRT modeling analysis results indicate that the optimal model fit is achieved when antisocial personality is set at four dimensions (AIC = 10858.560; BIC = 11803.640; LogLik = -5149.281; = 177.036; df = 31; RMSEA = .057; TLI = .914; CFI = .946).

This outcome supports the multi-dimensional approach to assessing antisocial personality traits, aligning with our goal of developing a comprehensive and reliable gamified assessment tool (ASP-ECD-G). The low RMSEA and high TLI and CFI values further validate the stability and reliability of the four-dimensional model. These findings indicate that the gamified assessment tool not only captures the complexity of antisocial personality traits effectively but also provides a robust framework for practical application. Therefore, the successful application of IRT modeling, despite theoretical concerns, enhances the credibility and utility of the ASP-ECD-G tool in psychological research, organizational recruitment, and employee management. This comprehensive validation underscores the tool's effectiveness and its potential for broader adoption in diverse contexts.
